# MicroRNA miR-4779 suppresses tumor growth by inducing apoptosis and cell cycle arrest through direct targeting of PAK2 and CCND3

**DOI:** 10.1038/s41419-017-0100-x

**Published:** 2018-01-23

**Authors:** Kyung Hee Koo, Heechung Kwon

**Affiliations:** Division of Basic Radiation Bioscience, Korea Institute of Radiological and Medical Sciences, Nowon-gil 75, Nowon-gu, Seoul 139-706 Republic of Korea

## Abstract

Depending on the function of their target genes, microRNAs (miRNAs) act as either tumor suppressors or oncogenes. Therefore, miRNAs represent a novel therapeutic strategy for prevention and management of cancer by targeting of onco-miRNAs or mimicking of tumor suppressor miRNAs. Herein, we identified novel tumor suppressor miRNAs and investigated their molecular mechanisms. To identify novel tumor suppressor miRNAs, we used 532 human miRNA mimic libraries and measured cell viability using MTS assays. The function of miR-4779 was then analyzed using cell cycle analyses and apoptosis, colony forming, and soft agar assays. Target genes of miR-4779 were predicted using TargetScan and miRDB databases and were confirmed using luciferase assays. Levels of miR-4779 and target genes in colon cancer tissue samples from patients were evaluated using qRT-PCR and western blotting analyses. Finally, in vivo tumor suppressive effects of miR-4779 were evaluated in HCT116 xenografts. In this study, miR-4779 inhibited cancer cell growth by inducing apoptosis and cell cycle arrest, and the putative survival factors PAK2 and CCND3 were identified as direct targets of miR-4779. In subsequent experiments, PAK2 knockdown induced cell cycle arrest and CCND3 knockdown induced cell cycle arrest and apoptosis. In addition, miR-4779 suppressed tumor growth and tumorigenesis in an in vivo HCT116 xenograft model. Finally, miR-4779 expression was low in 9 of 10 colon cancer tissues, whereas PAK2 and CCND3 expressions were significantly high in colon cancer tissues. The novel tumor suppressor miR-4779 inhibits cancer cell growth via cell cycle arrest and apoptosis by directly targeting PAK2 and CCND3. The present data indicate the potential of miR-4779 as a therapeutic target for miRNA-based cancer therapy.

## Introduction

MicroRNAs (miRNAs) are a class of endogenous, small non-coding RNA molecules that bind to 3′-untranslated regions (3′UTR) of target genes and block translation, leading to target mRNA degradation and inhibited expression of various target genes^1^. By targeting multiple transcripts, miRNAs are involved in diverse biological processes, including proliferation, development, differentiation, and apoptosis. Moreover, because they control biological processes that are implicated in carcinogenesis, miRNAs have been linked to cancer development^2–4^. Depending on targeted genes, miRNAs can be considered as tumor suppressors or oncogenes^5–7^. Aberrant expression of miRNAs has been shown in various types of cancer, including breast, lung, prostate, and colon cancers^8–11^. Therefore, miRNAs represent a novel therapeutic strategy for the prevention and management of cancer, involving targeting of onco-miRNAs or mimicking of tumor suppressor miRNAs.

Growing evidence shows that tumor suppressor miRNAs can be used as effective cancer therapies because they are often downregulated in cancer tissues. For example, miR-34a is a well-defined tumor suppressor miRNA that regulates the p53 pathway and inhibits cancer cell growth by directly targeting oncogenes such as Myc, c-Met, Bcl-2, CDK4, CDK6, cyclin D1, and cyclin E1^12,13^. Moreover, miR-34a is downregulated in numerous cancers and is known as a master tumor suppressor because of its broad anti-oncogenic activity. Hence, miR-34a could be exploited using novel anticancer drugs effective against heterogeneous tumors^14^. Accordingly, a current clinical trial of the miR-34 mimic MRX34 is being conducted in primary liver cancer, lymphoma, melanoma, multiple myeloma, renal cell carcinoma, small cell lung carcinoma, and non-small cell lung carcinoma (Mirna Therapeutics, Austin, TX, NCT01829971).

Numerous novel miRNAs have been identified after their initial discovery, and in January 2017, 2588 mature human miRNA sequences had been deposited in the central repository of miRNA sequences miRBase (release 21). Therefore, intensive screening of newly discovered miRNAs is required to identify effective tumor suppressor miRNAs. Herein, we screened a miRNA library and identified miR-4779 as a novel tumor suppressor, and elucidated the mechanisms by which miR-4779 suppresses cancer cell growth. Specifically, we investigated the role of miR-4779 in colon cancer and identified direct target genes that are involved in apoptosis and cell cycle arrest. Finally, we showed that miR-4779 negatively regulates the expression of the oncogenes PAK2 and CCND3, further suggesting that miR-4779 acts as a tumor suppressor.

## Results

### Screening of tumor suppressive miRNAs

To identify novel tumor suppressor miRNAs, we used 532 miRNA mimic libraries (Supplementary Table 1) that included the most recently discovered miRNAs with currently unknown functions. In our initial screening, 532 miRNAs were transfected into HCT116 colon cancer cells and the cell viability was determined using MTS assays. In further experiments, we chose the 30 miRNAs with the highest anti-proliferative effects in HCT116 cells (Supplementary Fig. 1) and determined their effects on cell viability in A549 and H460 lung cancer cells, MCF-7 breast cancer cells, and HT-29 colon cancer cells (Supplementary Fig. 2). The resulting data show differential effects of these 30 miRNAs on the viability of various cancer cells. Among the 30 miRNAs, miR-4779 significantly inhibited cell viability in all cancer cells and was selected for further studies.

### miR-4779 inhibits tumor cell growth by inducing apoptosis and cell cycle arrest

**Fig. 1:**
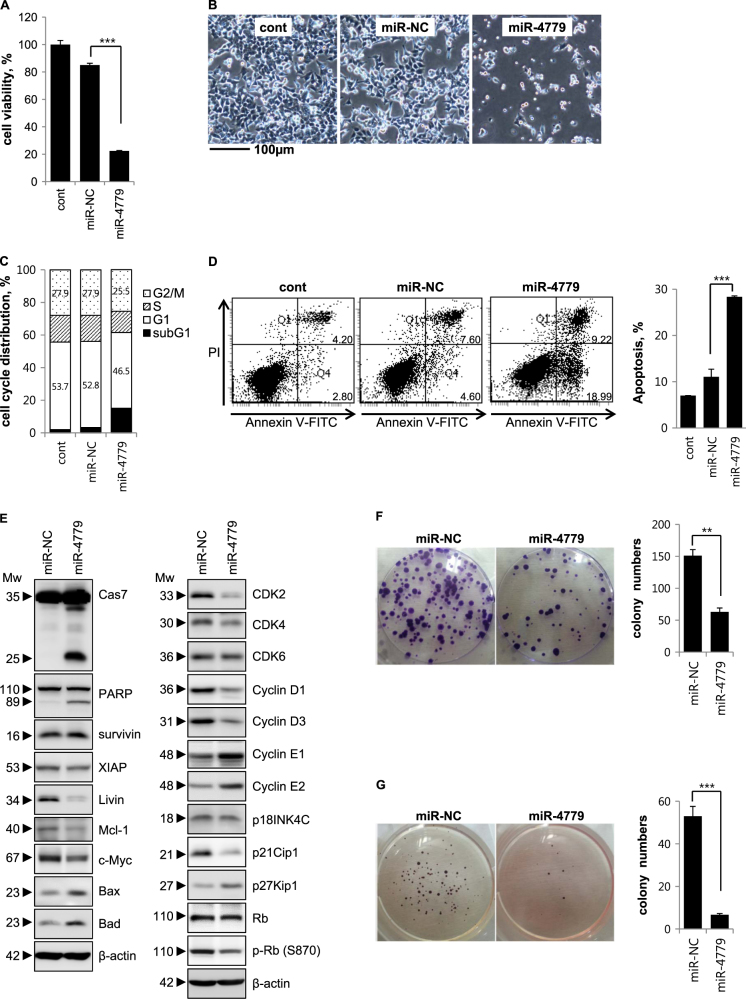
miR-4779 inhibits tumor cell growth and induces apoptosis **a–g** HCT116 cells were transfected with miR-NC or miR-4779. At 72 h after transfection, cell viability was determined by MTS assay **a**. The non-transfected control (cont) was normalized to 100% cell viability and the effects of the miR-NC or miR-4779 were calculated accordingly. At 48 h after transfection, cell images were captured using phase contrast microscopy **b**. Magnification = ×100, scale bar = 100 μm. Cell cycle was analyzed by flow cytometry **c** and the percentage of cells in G1 and G2/M phase are annotated in each column. Apoptosis was measured by flow cytometric analysis of cells stained with Annexin V-FITC and PI **d**. The percentages of Annexin V-FITC only positive cells (right lower, early apoptosis) and both Annexin V-FITC and PI positive cells (right upper, late apoptosis) were quantitated. Right, the quantified apoptotic cell population was estimated. Equal amounts of cell lysates were subjected to western blotting using the indicated antibodies **e**. β-actin was used as a loading control. At 24 h after transfection, cells were re-plated and performed colony formation **f** and soft agar assays **g**. Each colony was stained, taken (left), and counted (right). Data are presented as averages of triplicate measurements with error bars representing standard deviations. **P < 0.01 and ***P < 0.001. All experiments were performed tree times on separate samples with comparable results

To confirm the role of miR-4779 as a tumor suppressor, we analyzed cell viability (Fig. 1a), morphology (Fig. 1b), cell cycle stages (Fig. 1c), and apoptotic cell populations (Fig. 1d). Cell viability was significantly decreased by 74% in miR-4779-transfected cells compared with miR-NC-transfected cells (Fig. 1a–b). Cell cycle analyses showed that miR-4779 increased numbers of cells in the subG1 population and subsequently caused apoptosis (Fig. 1c). Increased apoptotic cell populations were also observed among miR-4779-transfected cells, with >2.5-fold increases in apoptotic cell numbers than that among miR-NC-transfected cells (Fig. 1d). These results clearly suggest that miR-4779 exerts tumor suppressive effects by inducing cell cycle arrest at the G1 phase and apoptosis. In addition, miR-4779-induced apoptosis was accompanied by caspase-7 activation and PARP cleavage (Fig. 1e) and the pro-apoptotic proteins Bax and Bad were induced by miR-4779 without changes in the levels of anti-apoptotic proteins such as survivin. Anti-apoptotic proteins and oncogenes such as XIAP, Mcl-1, Livin, and c-Myc, were also slightly or significantly decreased in miR-4779 expressing cells (Fig. 1e). These results indicate that miR-4779 promotes apoptosis by downregulating Mcl-1, Livin, and c-Myc, and inducing the accumulation of Bax and Bad. Furthermore, following transfection with miR-4779, PARP cleavage and cell death or cell growth inhibition were observed in various cancer cells (Supplementary Fig. 3).

To specifically identify the cell cycle regulators that mediate G1 arrest after induction by miR-4779, we performed Western blot analysis using antibodies against cyclins, cyclin dependent kinases (CDKs), and CDK inhibitors. Key regulators of G1 progression include the D-type cyclins Dl, D2, and D3, which assemble into holoenzymes with either CDK4 or CDK6, and cyclin E, which binds CDK2 during the late G1 phase. Cyclin D-CDK4/6 and cyclin E-CDK2 complexes phosphorylate the retinoblastoma (Rb) protein to induce cell cycle progression from G1 to S phases. CDK inhibitors such as p27Kip1, p21Cip1, and members of the INK4 family (p16INK4A, p15INK4B, p18INK4C, and p19INK4D) can bind cyclin D-CDK4/6 or cyclin E-CDK2 complexes and inhibit their CDK activity, leading to G1 arrest. As shown in Fig. 1e, cyclin D1, cyclin D3, and CDK4 levels were markedly decreased in miR-4779-transfected cells compared with miR-NC-transfected cells, whereas no changes in CDK6 levels were observed. Cyclin E1 and cyclin E2 expression levels were also significantly increased, whereas CDK2 expression was significantly decreased. In addition, following transfection with miR-4779, phospho-Rb (S870) protein levels were decreased without changes in the expression of the Rb protein. Moreover, the CDK inhibitor p27Kip1 was significantly induced, and the other CDK inhibitors p18INK4C and p21Cip1 had slightly and significantly decreased expression, respectively (Fig. 1e). The protein p27Kip1 acts as a critical negative regulator of the cell cycle by inhibiting the activity of cyclin/CDK complexes during G0 and G1 phases. Therefore, the present observations indicate that miR-4779 downregulates CDK2, CDK4, cyclin D1, and cyclin D3, and induces p27Kip1 accumulation, leading to inhibition of Rb phosphorylation and G1 arrest.

### miR-4779 inhibits clonogenic survival and anchorage-independent tumor growth

To evaluate the effects of miR-4779 on in vitro tumorigenesis, we measured clonogenic survival of miR-4779-transfected cells in colony-forming assays (Fig. 1f) and assessed anchorage-independent growth in soft agar assays (Fig. 1g). Clonogenic survival and anchorage-independent growth were significantly less in miR-4779-transfected cells than in miR-NC-transfected cells, with 3- and 5-fold differences, respectively (Fig. 1f, g). These results show that miR-4779 inhibits clonogenic survival and anchorage-independent growth of tumor cells, suggesting that miR-4779 plays a crucial role in tumorigenesis.

### miR-4779 directly targets to PAK2 and CCND3

To investigate tumor suppressive mechanisms of miR-4779, candidate miR-4779 target genes of were analyzed using the miRNA target prediction programs TargetScan (http://www.targetscan.org) and miRDB (http://mirdb.org/miRDB). These computations revealed two and one putative miR-4779 binding sites in the 3′UTR of p21-activated kinase 2 (PAK2) and cyclin D3 (CCND3) mRNAs, respectively (Supplementary Fig. 4). Previous studies show that PAK2 promotes cancer cell survival and is overexpressed in various cancers. Similarly, cyclin D3 is critical for cancer cell growth, proliferation, and development, and is highly expressed in many human cancers.

**Fig. 2:**
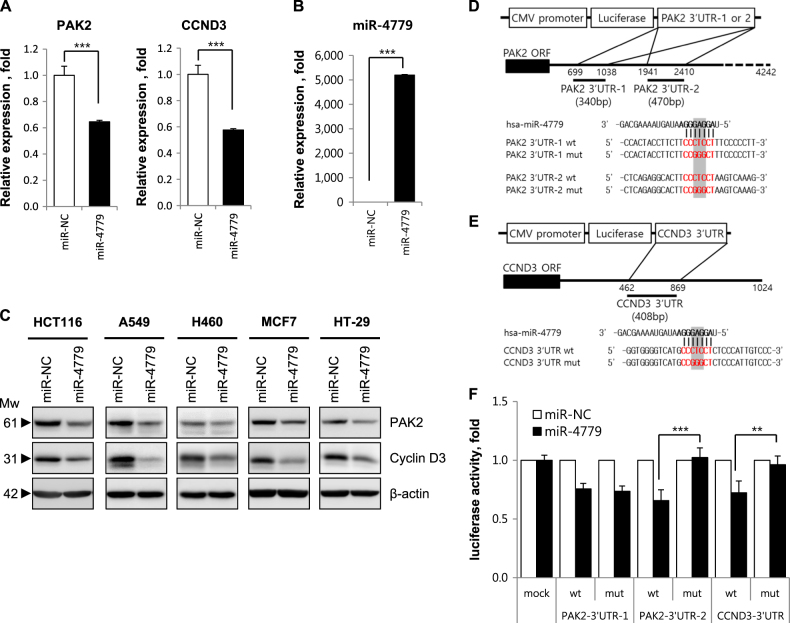
miR-4779 down-regulates PAK2 and CCND3 by direct targeting **a**, **b** HCT116 cells were transfected with miR-NC or miR-4779 for 24 h. The mRNA levels of PAK2 and CCND3 were determined by qRT-PCR **a**. HPRT1 was used as a normalizer. Data show the relative each gene expression in miR-4779 transfected samples as compared with a miR-NC transfected samples. The expression of mature miR-4779 was determined by qRT-PCR **b**. U6 snRNA was used as a normalizer. **c** HCT116, A549, H460, MCF7, and HT-29 cells were transfected with miR-NC or miR-4779 for 48 h, and the protein levels of PAK2 and CCND3 were determined by western blotting using the corresponding antibodies. **d-e** Structure of reporter constructs containing 3’UTRs of PAK2 **d** and CCND3 **e** downstream of the luciferase ORF. The aligned sequences of constructs (red) complementary to the seed sequence of the miR-4779 (bold) and the mutant sequences are shown. The mutated regions are shaded. HCT116 cells were transfected with a combination of the indicated reporters and miRNAs, and luciferase assay was performed 48 h after transfection **f**. The fold change in relative luciferase activity in transfected cells with miR-NC was set as 1. Data are presented as averages of triplicate measurements with error bars representing standard deviations. ^**^P < 0.01 and ^***^P < 0.001

To determine whether miR-4779 represses these putative targets, HCT116 cells were transfected with miR-4779 and mRNA and protein levels of target genes were assessed using qRT-PCR and western blotting analyses, respectively (Fig. 2a–c). Increased miR-4779 expression significantly decreased PAK2 and CCND3 mRNA expression in HCT116 cells (Fig. 2a), and PAK2 and CCND3 protein levels were correspondingly lower than in miR-NC-transfected cells (Fig. 2c). The efficiency of miR-4779 transfection was checked by qRT-PCR (Fig. 2b). Additionally, transfection with miR-4779 led to significant reductions in PAK2 and CCND3 protein expression levels in A549, H460, MCF7, and HT-29 cancer cell lines (Fig. 2c). These data indicate that miR-4779 negatively regulates PAK2 and CCND3 at both transcriptional and translational levels in various cancer cells.

To determine whether miR-4779 directly targets PAK2, we performed target predictions using TargetScan and found two putative binding sites (position of 894–900 and 2106–2112) in the 3′UTR that are conserved in primates (Supplementary Fig. 4a). As shown Fig. 2d, two PAK2 3′UTR fragments (PAK2 3′UTR-1 and PAK2 3′UTR-2) containing wild-type or mutant sequences were cloned into the pGL3-luc vector downstream of the firefly luciferase reporter gene. Luciferase activities of both wild type and mutant PAK2 3′UTR-1 constructs were decreased by 25% in miR-4779-transfected cells compared with miR-NC-transfected cells (Fig. 2f). Moreover, whereas the luciferase activity of the PAK2 3′UTR-2 wild-type construct was decreased by 35% in miR-4779-transfected cells, that of the PAK2 3′UTR-2 mutant construct was not suppressed in comparison with miR-NC-transfected cells (Fig. 2f). These results indicate that miR-4779 directly binds to the PAK2 3′UTR-2 but not to the PAK2 3′UTR-1. CCND3 is another potential target of miR-4779 that contains one miR-4779 target site (position of 795–801) in its 3′UTR, which is broadly conserved among mammals (Supplementary Fig. 4b). After cloning CCND3 3′UTR fragments containing wild-type or mutant miR-4779 binding sequences into the pGL3-luc vector downstream of the firefly luciferase reporter gene (Fig. 2e), luciferase activity of the reporter containing the CCND3 3′UTR was suppressed by 30% following transfection with miR-4779 (Fig. 2f). However, luciferase activities of the mutant reporter construct were not repressed (Fig. 2f). These results indicating that miR-4779 targets PAK2 and CCND3 directly.

### PAK2 and CCND3 expression and tumor cell growth are not suppressed by an inhibitor of miR-4779

**Fig. 3:**
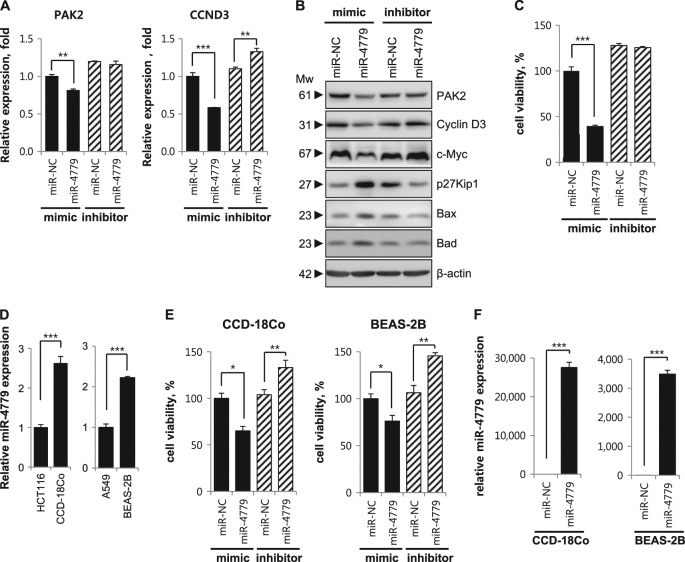
miR-4779 expression and cell viability by miR-4779 inhibitor are higher in normal cell lines than cancer cell lines **a**–**c** HCT116 cells were transfected with mimics or inhibitors of miR-NC and miR-4779. At 24 h after transfection, mRNA expression levels of PAK2 and CCND3 were determined by qRT-PCR **a**. At 48 h after transfection, protein levels of PAK2 and CCND3 were determined by western blotting using the corresponding antibodies **b**. At 72 h after transfection, cell viability was determined by MTS assay **c**. **d** Comparison of miR-4779 expression in cancer and normal cells. Expression of mature miR-4779 was determined by qRT-PCR. e Normal cell lines CCD-18Co and BEAS-2B were transfected with mimics or inhibitors of miR-NC and miR-4779. At 72 h after transfection, cell viability was determined by MTS assay. **f** Normal cell lines CCD-18Co and BEAS-2B were transfected with miR-NC or miR-4779 for 24 h. The expression of mature miR-4779 was determined by qRT-PCR. All data are presented as averages of triplicate measurements with error bars representing standard deviations. **P < 0.01 and ***P < 0.001

To further characterize the suppressive effects of miR-4779 on PAK2 and CCND3 expression, we blocked the function of endogenous miR-4779 using antisense oligonucleotides (miR-4779 inhibitor). Under these conditions, PAK2 and CCND3 protein and mRNA levels were not suppressed by miR-4779 inhibitor (Fig. 3a, b), and CCND3 expression levels were slightly increased. In agreement, cell viability, apoptosis, clonogenic survival, and anchorage-independent cell growth were not significantly affected in the presence of the miR-4779 inhibitor compared with those in the presence of the miR-NC inhibitor (Fig. 3c and Supplementary Fig. 5). Moreover, miR-4779 mediated induction of p27Kip1, Bax, and Bad as well as c-Myc downregulation were suppressed by the miR-4779 inhibitor (Fig. 3b), and in contrast with the miR-4779 mimic, the miR-4779 inhibitor downregulated p27Kip1 and Bax and upregulated c-Myc. These results show that miR-4779 inhibitor blocks the function of endogenous miR-4779, although these effects were limited by low endogenous miR-4779 levels in HCT116 cells.

### miR-4779 expression and cell viability by miR-4779 inhibitor are higher in normal cell lines than cancer cell lines

To test the possibility of miR-4779 as a tumor suppressor, we compared miR-4779 expression between cancer and normal cell lines from the same tissue of origin. The human normal colon fibroblast cell line CCD-18Co exhibited significantly higher miR-4779 expression than the colon cancer cell line HCT116. In addition, the human normal lung epithelial cell line BEAS-2B expressed higher levels of miR-4779 than the lung cancer cell line A549 (Fig. 3d). Next, we examined whether miR-4779 affects the cell viability of normal cells. We found that the cell viability of CCD-18Co and BEAS-2B cells was higher than that of cancer cells when exposed to miR-4779 mimics (Fig. 3e). We then examined the effect of inhibition of endogenous miR-4779 activity on the cell viability of miR-4779-high normal cell lines. Transfection with a miR-4779 inhibitor significantly enhanced the cell viability of both CCD-18Co and BEAS-2B cells compared with that with a miR-NC inhibitor (Fig. 3e). The transfection efficiency of miR-4779 in CCD-18Co and BEAS-2B cells was checked by qRT-PCR (Fig. 3f). These results suggest that miR-4779 is more effective as a tumor suppressor in cancer cells than normal cells.

### Knockdown of PAK2 or CCND3 inhibits tumor cell growth

**Fig. 4:**
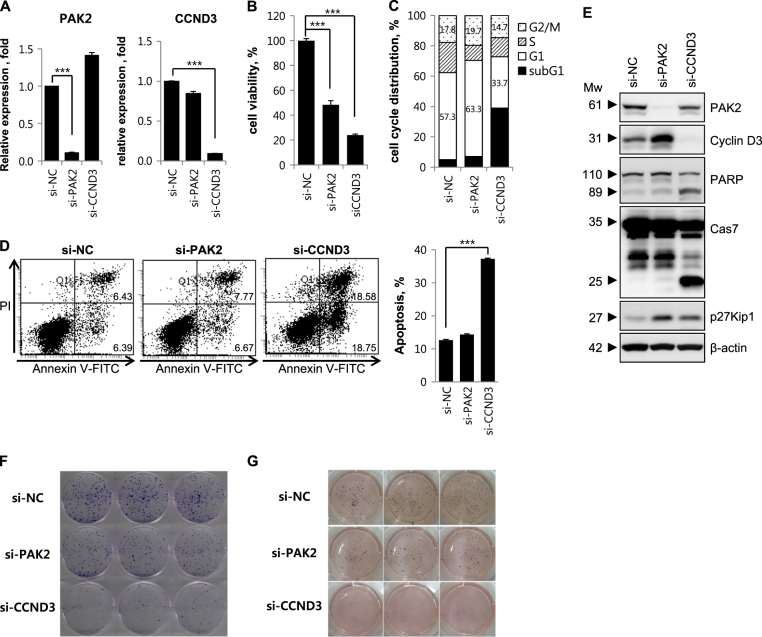
Knockdown of PAK2 or CCND3 inhibits tumor cell growth **a–g** HCT116 cells were transfected with si-RNAs for negative control (si-NC), PAK2 (si-PAK2), or CCND3 (si-CCND3). At 24 h after transfection, mRNA levels of PAK2 and CCND3 were determined by qRT-PCR **a**. At 72 h after transfection, cell viability was determined by MTS assay **b**. At 48 h after transfection, cell cycle was analyzed **c** and the percentage of cells in G1 and G2/M phase are annotated in each column. At 48 h after transfection, apoptosis was measured by flow cytometric analysis of cells stained with Annexin V-FITC and PI **d**. Right, the quantified apoptotic cell population was estimated At 48 h after transfection, equal amounts of cell lysates were subjected to western blotting using the indicated antibodies **e**. At 24 h after transfection, cells were re-plated and performed colony formation **f** and soft agar assays **g**. All data are presented as averages of triplicate measurements with error bars representing standard deviations. ***P < 0.001

To determine whether miR-4779 inhibits cell growth by targeting PAK2 or CCND3 encoding genes, we performed RNA interference (RNAi) experiments. PAK2 and CCND3 knockdown was confirmed in qRT-PCR and Western blotting analyses, which showed marked reductions in mRNA and protein levels (Fig. 4a–e). PAK2 and CCND3 knockdown suppressed cell viability by 52% and 76%, respectively (Fig. 4b). Accordingly, clonogenic survival and anchorage-dependent growth were moderately and highly decreased by si-PAK2 and si-CCND3, respectively (Fig. 4f, g), indicating that miR-4779 inhibits cell growth by targeting both PAK2 and CCND3. These effects were confirmed in investigations of cell cycle arrest and apoptosis, in which si-PAK2 induced cell cycle arrest at G1 and G2/M phases but did not induce apoptosis, and si-CCND3 induced cell cycle arrest at the G1 phase, leading to increased numbers of apoptotic cells and increased caspase-7 activation and PARP cleavage (Fig. 4c–e). Moreover, significant p27Kip1 accumulation was observed in the presence of si-PAK2 or si-CCND3 (Fig. 4e). These data further show that miR-4779 induces cell cycle arrest by targeting PAK2 and CCND3, leading to accumulation of p27Kip1 and increased apoptosis.

### miR-4779 inhibits tumor growth in a mouse xenograft models

**Fig. 5:**
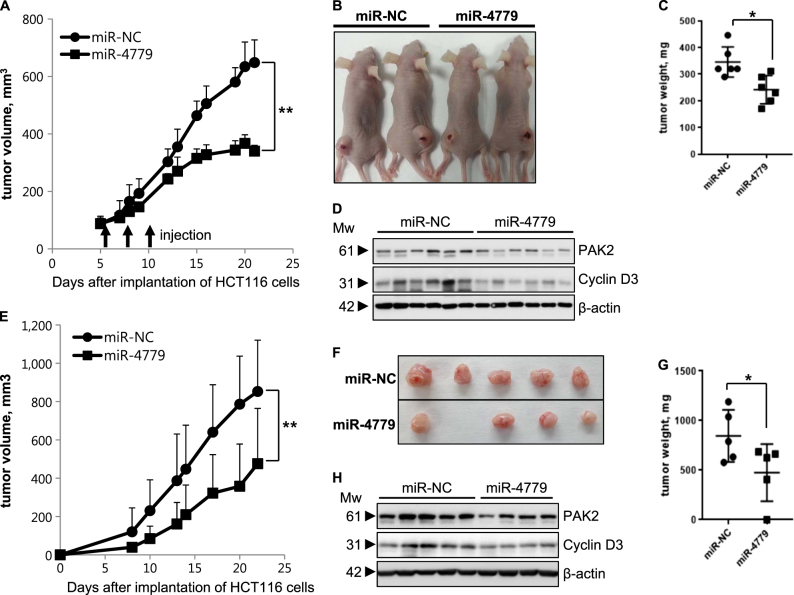
miR-4779 inhibits tumor growth in a mouse xenograft models **a**–**d** Intratumoral injection of miR-4779 suppresses tumor growth in mouse xenografts (*n* = 6). **a** HCT116 cells were injected into mice with subcutaneously and miR-NC or miR -4779 was injected as a complex with in vivo-jetPEI into tumors at the indicated time points (arrows), and tumor sizes were measured at the indicated intervals and plotted. **b** Representative images showing the sizes of tumors injected with miR-NC or miR-4779 at 22 days in **A**. **c** At 22 days in A, tumors dissected from mice of each group were weighted and plotted. **d** Tumor tissues were performed western blot analysis using indicated antibodies. **e-h** miR-4779 inhibits subcutaneous tumor growth in mouse xenografts (*n* = 5). **e** HCT116 cells from transfected with miR-NC or miR-4779 were injected into mice subcutaneously and tumor sizes were measured at the indicated intervals and plotted. **f** Representative images of the tumor-bearing mice and removed tumors. Mice were sacrificed at 22 days in **e**. **g** Tumors dissected from mice of each group (in **f**) were weighed and plotted. **h** Tumor tissues were performed western blot analysis using indicated antibodies. Error bars represent mean ± SEM. *P < 0.05 and **P < 0.01

To determine in vivo tumor suppressive effects of miR-4779, we increased miR-4779 expression in tumor cells of mouse xenografts using two experimental approaches. Initially, we injected miR-4779 or miR-NC in complex with an in vivo transfection agent into established HCT116 tumor xenografts. After three injections of miR-4779 at 3-day intervals, tumor growth was significantly lower than that following injection of miR-NC (Fig. 5a–d). In subsequent experiments, HCT116 cells were transfected with miR-4779 or miR-NC before subcutaneous implantation and were then established as xenograft tumors. In agreement with in situ tumor transfection experiments, miR-4779 expressing tumors grew significantly more slowly than miR-NC expressing tumors (Fig. 5e–h). In addition, western blot analysis revealed that both PAK2 and cyclin D3 were downregulated in the miR-4779 expressed tumors (Fig. 5d–h). Taken together, these results clearly demonstrate that miR-4779 functions as a tumor suppressor in vivo, and suggest high potential for application as an anticancer therapy.

### Level of miR-4779 is low in colon cancer tissues, whereas PAK2 and CCND3 were highly expressed

**Fig. 6:**
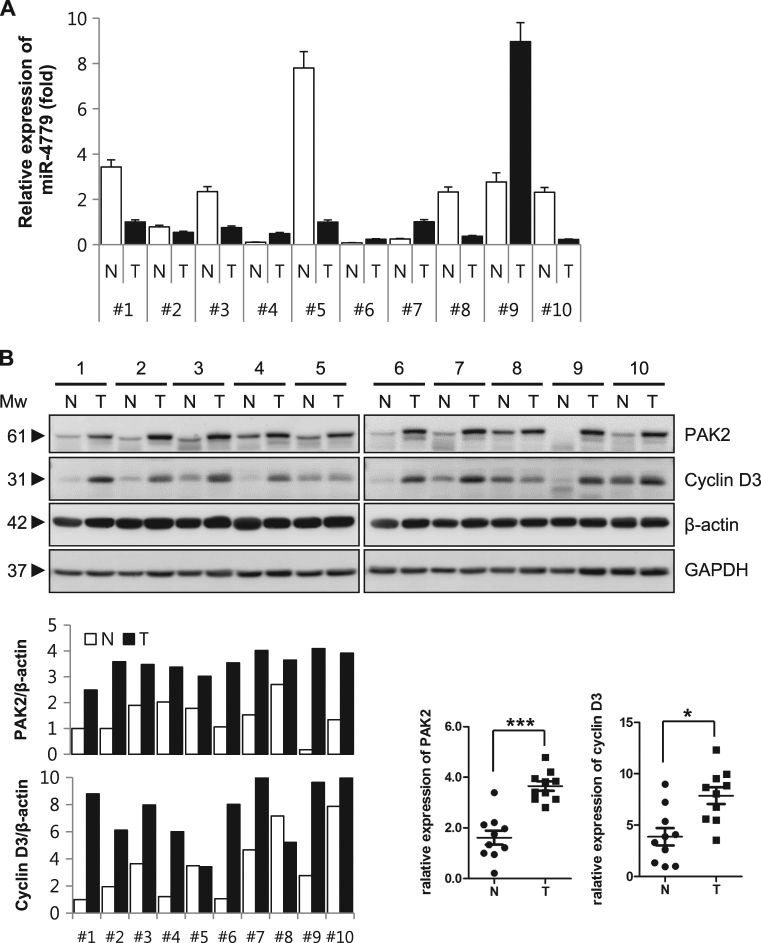
Level of miR-4779 was low in colon cancer tissue, whereas expression of PAK2 and CCND3 were highly up-reregulated **a** Comparison of miR-4779 expression in tumor (T) and adjacent normal (N) tissues from colon cancer patients. Small RNA was extracted from each tissue and qRT-PCR was performed. U6 snRNA was used as a normalizer. 10 pairs of frozen tumor and adjacent normal tissues from colon cancer patient were used in this experiment. The expression level of miR-4779 in tumor tissue of patient #1 was set at 1, and the relative amounts of miR-4779 at the other samples were plotted as fold induction. Error bars represent mean ± SEM. **b** Comparison of protein expression of PAK2 and CCND3 in tumor (T) and adjacent normal (N) tissues from colon cancer patients. Equal amount of tissue lysates were subjected to western blotting using the corresponding antibodies. β-actin and GAPDH were used as a loading control. The levels of PAK2 and CCND3 were quantified by densitometry and normalized to β-actin levels. The ratios of PAK2 or CCND3 to total β-actin in normal tissue of patient #1 were set at 1

To validate the clinical relevance of miR-4779 as a tumor suppressor, we determined miR-4779 expression and its target proteins in tumors and adjacent normal tissues from colon cancer patients. In these experiments, tissue lysates and small RNA samples were prepared from 10 pairs of frozen normal and tumor tissues, and the expression of mature miR-4779 was determined using qRT-PCR. These analyses showed higher miR-4779 expression in normal tissues than in tumor tissues, with >2.5-fold differences in 5 of 10 pairs. In 9 of 10 tumors, miR-4779 levels were exceedingly low (Fig. 6a). Accordingly, PAK2 and CCND3 expression levels were markedly higher in tumor tissues than in normal tissues (Fig. 6b). Taken together, these data suggest that miR-4779 mimics have therapeutic potential as an anticancer therapy.

## Discussion

In this study, we performed functional screening analyses of 532 miRNA mimic libraries as a strategy for discovering novel miRNAs that suppress cancer cell growth. These analyses revealed 30 miRNAs that inhibit the growth of HCT116 cells, and these were more effective than the previously described miR-34a^14^. Among these miRNAS, we identified and characterized miR-4779 as a novel tumor suppressive miRNA that induces apoptosis and cell cycle arrest in cancer cells and then assessed its potential as a candidate anticancer therapy. Mature miR-4779 was previously identified in breast cancers^15^ and was co-localized with Transcription Factor 7 Like 1 (TCF7L1) within the same common fragile sites, which are vulnerable structural domains that may be functionally sensitive to replication stress^16^.

In the present study, miR-4779 effectively inhibited the growth of HCT116 cells and other cancer cell lines. Thus, to explore the related molecular mechanisms, we predicted miR-4779 target genes using TargetScan and miRDB databases and identified two targets that were responsible for apoptosis inducing and growth inhibiting actions of miR-4779. The first of these, PAK2, is a serine/threonine protein kinase with reported physiological roles in motility, survival, mitosis, and apoptosis^17^. Proteins of the PAK family were initially identified as binding partners of the Rho GTPases Cdc42 and Rac1^18^ and are well-known regulators of cytoskeletal remodeling and cell motility^19^. Accumulating evidence indicates that PAK2 overexpression is correlated with disease progression in human cancers^20–22^. Moreover, alterations in PAK expression have been detected in human tumors, suggesting that PAK could be an attractive new therapeutic target^23–25^ and that direct PAK2 downregulation by miR-4779 induces cell cycle arrest. Accordingly, knockdown of PAK2 induced cell cycle arrest at the G1 phase and caused p27Kip1 accumulation (Fig. 4c–e). In a previous study, two miRNAs were shown to directly target PAK2, and among these, miR-137 inhibited proliferation in melanoma cells^26^. In addition, miR-134 was shown to mediate Bad phosphorylation, and affected cell survival and apoptosis by targeting PAK2 in ovarian-cancer cells^27^. In agreement, repression of miR-134 and consequent upregulation of PAK2 contributed to paclitaxel resistance in these ovarian cancer cells^27^. Activation of full-length PAK2 promotes cell survival through the phosphorylation of Bad^28^, whereas proteolytic activation of PAK2 p34 leads to apoptosis^29^. The present findings show that neither treatment with a miR-4779 mimic nor PAK2 knockdown decreased Bad phosphorylation levels in HCT116 cells (Supplementary Fig. 6). Elevated PAK2 activity reportedly interrupts apoptotic responses, causes anchorage-independent survival and growth, and leads to resistance of breast cancer cells to drug-induced apoptosis^30^. Moreover, PAK2 was shown to inhibit chemotherapeutic drug-induced apoptosis by phosphorylating caspase-7 in breast cancer cells^31^. Hence, PAK2 downregulation by miR-4779 may inhibit chemo-resistance in various cancers, although further studies are required to confirm the clinical efficacy of miR-4779 mimics.

In the present experiments, miR-4779 induced cell cycle arrest and apoptosis by directly targeting cyclin D3. Key regulators of G1 progression include cyclins D1, D2, and D3, which form complexes with CDK4 or CDK6 and induce hyper-phosphorylation of the Rb protein. Phosphorylation of Rb is required for cell cycle progression from G1 to S phases^32^ and cyclin D is frequently deregulated in cancer and is considered a biomarker of cancer phenotypes and disease progression^33^. CCND3 is reportedly expressed at high levels in most human cancers, including colorectal cancer (CRC)^34^. Herein, CCND3 and phospho-Rb were downregulated in miR-4779 transfected HCT116 cells (Figs. 1e and 2c). Furthermore, CCND3 knockdown in HCT116 cells negatively affected cell proliferation (Fig. 4), suggesting that miR-4779 directly targets CCND3 to inhibit cancer cell proliferation. In accordance, CCND3 is critical for cell growth, proliferation, and cancer development, and is directly targeted by various miRNAs. Specifically, miR-195 regulated glioblastoma cell invasion by modulating the CCND3/p27Kip1 pathway^35^ and miR-503 inhibited the G1/S transition by targeting CCND3 and E2F3 in hepatocellular carcinomas^36^. Moreover, miR-138 reportedly induced cell cycle arrest by targeting CCND3 in hepatocellular carcinoma cells^37^ and miR-592 inhibited cell proliferation by suppressing CCND3 expression in CRC^38^ Similarly, miR-4779 efficiently induced cell cycle arrest and apoptosis by targeting CCND3, and further contributed to cell cycle arrest by suppressing PAK2 expression.

No database for miR-4779 expression in colon cancer is available in public genomics databases. Thus, we analyzed miR-4779 expression levels and its target proteins in 10 paired clinical samples of normal and cancer tissues. In these frozen tissue specimens from colon cancer patients, lower miR-4779 expression was observed in 6 of 10 pairs of tumors compared with normal tissues, whereas PAK2 and CCNCD3 proteins were highly upregulated in tumors (Fig. 6). The bioinformatics database MIRUMIR shows that the high expression of miR-4779 results in a lower survival rate in esophageal squamous cell carcinoma patients, which was not significant (*p* = 0.611) (Supplementary Fig. 7)^39^. This data indicates that the level of miR-4779 expression has little relevance to overall survival in cancer. The Pearson correlation analysis showed that miR-4779 expression had no significant correlation with PAK2 or CCND3 expression (Supplementary Fig. 8). However, miR-4779 may have potential as a tumor suppressor in cancer patients with low miR-4779 and high PAK2/CCND3 expressions. Corresponding miR-4779 expression levels and its target proteins were also observed in various cancer and normal cell lines (Supplementary Fig. 9), and an inhibitor of miR-4779 blocked the function of endogenous miR-4779 in HCT116, CCD-18Co, and BEAS-2B cells (Fig. 3c–e). MiR-4779 expression was higher in normal cell lines, such as CCD-18Co and BEAS-2B, than in cancer cell lines, such as HCT116 and A549 (Fig. 3d). Also, normal cell lines with high miR-4779 expression were less affected by exogenous miR-4779 compared with cancer cell lines but were more affected by a miR-4779 inhibitor (Fig. 3c–e). These data indicate that the effect of miR-4779 on cell viability is dependent on endogenous miR-4779 levels. Finally, miR-4779 suppressed the growth of HCT116 xenograft tumors (Fig. 5), as indicated by decreased tumor growth and tumor sizes compared with controls. Advantages of therapeutic miRNAs include efficacy against heterogeneous tumors, reflecting simultaneous targeting of multiple cancer related proteins. Therefore, further studies of miR-4779 functions and investigations of target genes are warranted.

In this study, we identified miR-4779 as a novel tumor suppressor miRNA and demonstrated that miR-4779 induces cancer cell apoptosis and cell cycle arrest by targeting PAK2 and CCND3 in vitro and in vivo. To our knowledge, this is the first report to demonstrate the function of miR-4779, and our data suggest that miR-4779 has high potential as a potent tumor suppressor miRNA that can be exploited in the development of novel anticancer miRNA therapeutics.

## Materials and methods

### Cell culture

Human colon cancer cell lines HCT116 and HT-29, human lung cancer cell lines A549 and H460, and human breast cancer cell line MCF-7 were obtained from the American Type Culture Collection (ATCC, Rockville, MD, USA). Human normal lung epithelial cell line BEAS-2B and human normal colon fibroblast cell line CCD-18Co was obtained from the ATCC. HCT116, A549, H460, and BEAS-2B cells were maintained in RPMI1640 media (Welgene, Daegu, Korea) supplemented with 10% (v/v) fetal bovine serum (FBS) (Hyclone, Rockford, IL) and 1% (v/v) penicillin and streptomycin (Invitrogen, San Diego, CA). HT29 and MCF-7 cells were maintained in DMEM media (Welgene) supplemented with 10% (v/v) FBS and 1% (v/v) penicillin and streptomycin. CCD-18Co cells were maintained in MEM media (Welgene) supplemented with 10% (v/v) FBS and 1% (v/v) penicillin and streptomycin.

### Screening of miRNA libraries

Human mature miRNA mimic libraries (532 miRNA mimics, Supplementary Table 1) were purchased from Genolution (Seoul, Korea). Synthetic miRNA mimics were prepared as RNA duplexes designed on the base of miRDB sequences. HCT116 cells were plated into 96-well plate (2 × 10^3^ cells/well) and transfected with 50 nM miRNA mimics using G-Fectin (Genolution) by reverse transfection according to the manufacturer’s protocol. After 72 h transfection, cell viability was analyzed by MTS assay.

### MTS assay

We seeded cells into 96-well plates and transfected them with each of the miRNAs. Cell viability was measured after 72 h with the CellTiter96 Aqueous Non-radioactive Assay (Promega, Madison, WI), as described in the manufacturer’s instruction. Briefly, 20 μl of the MTS solution was added to each well, and the plates were incubated for another 2 h. The absorbance at 490 nm was measured by microplate reader (Multiskan EX, Thermo LabSystems, Champaign, IL).

### RNA oligoribonucleotides and transfection

Synthetic miRNA mimics were prepared by Genolution as RNA duplexes designed from the sequence of miR-4779 (5′- UAGGAGGGAAUAGUAAAAGCAG-3′) and miR-NC (negative control with scrambled sequence) (5′- ACUCUAUCUGCACGCUGACUU-3′). Also, synthetic miR-4779 mimic and miR-NC mimic (SMC-2003) were synthesized as RNA duplexes by Bioneer (Daejeon, Korea). miR-4779 inhibitor and miR-NC inhibitor (SMC-2103) were synthesized as single-stranded RNA oligonucleotides by Bioneer. miRNA mimics and inhibitors from Bioneer were used for only Fig. 3 experiment. Validated small interfering RNA (si-RNA) duplex of human PAK2 (si-PAK2, 100266), human CCND3 (si-CCND3, 1027310), and the negative control with scrambled sequence (si-NC, SN1003) were purchased from Bioneer. For RNA interference, cells were transfected with 20 nM si-RNAs, 50 nM miRNA mimics, and 50 nM miRNA inhibitors using Lipofectamin RNAiMAX reagent (invitrogen) by reverse transfection according to the manufacturer’s protocol.

### Cell Cycle analysis

Cells were collected and washed with cold-PBS. In total 2 × 10^6^ cells were fixed with 70% ethanol and stored at 4 °C for overnight. Cells were rehydrated with PBS for 10 min at RT and then cells were stained with propidium iodide (PI) staining solution contained with 50 μg/ml PI (Sigma), 2 μg/ml DNase-free RNase A (Calbiochem, La Jolla, CA), and 0.2 % NP-40 (USB, Cleveland, OH) in PBS for 15 min at 37 °C. Stained cells were analyzed for cell cycle analysis in BD FACSCanto II (BD Biosciences, San Jose, CA) and the raw data were analyzed by BD FACSDiva 7.0 program (BD Bioscience).

### Apoptosis analysis

Apoptotic cells were determined by Annexin V-FITC/propidium iodide (PI) double staining using Annexin V-FITC Apoptosis Detection Kit (BioVision, Milpitas, CA) according to the manufacturer’s recommendation. Briefly, cells were collected and washed with cold-PBS. 5 × 10^5^ cells were stained with Annexin V-FITC and PI in binding buffer (10 mM HEPES, pH 7.4, 140 mM NaCl, 25 mM CaCl2) for 10 min. The stained cells were then analyzed using a BD FACSCanto II and the raw data were analyzed by BD FACSDiva 7.0 program.

### Colony-forming assay

After 24 h transfection, cells were collected, re-plated into 6-well plates (200 cells/well) and incubated for 7 day. Colonies were fixed and stained with 0.4% crystal violet (Bio Basic Inc., Markham, Canada) in 20% ethanol for 5 min. Cell colonies were photographed and counted.

### Soft agar assay

After 24 h transfection, cells were harvested and performed the soft agar assay for determination of the anchorage-independent growth. Briefly, plated out 1 ml of 0.8% agar (BD Bacto Agar, Sparks, MD) solution in culture media into 6-well plate and solidified, and then overlaid with 1 ml of 0.3% agar solution in culture medium containing 2000 cells and allowed to grow. To prevent dry, 0.5 ml culture medium was added to each well. After 14 day, 0.5 ml of iodonitrotetrazolium violet (INT, 0.5 mg/ml in PBS) (Sigma, St. Louis, MO) was added to stain the cells for overnight. Cell colonies were photographed and counted.

### Reporter assay

To prepare the reporter constructs, the 3′UTR of target genes containing the putative miR-4779 binding sites were amplified by PCR with the each indicated primers (Supplementary Table 2) and HCT116 cDNA as a template. The amplified PCR products were cloned the downstream of the luciferase gene in the pGL3-luc vector (kindly provided by V.N. Kim, School of Biological Sciences, Seoul National University, Korea) as schematically depicted in Fig. 2d, e. For generation of the mutant reporters, three nucleotide mutations were introduced into the putative miR-4779 binding sites using a QuikChange II XL site-directed mutagenesis kit (Agilent Technologies, Santa Clara, CA) with listed primers (Supplementary Table 2) according to the manufacturer’s recommendation. All primers were purchased from Bioneer. HCT116 cells were cotransfected with reporter plasmid (200 ng), pRL-CMV-Renilla plasmid (10 ng), and 10 nM miRNA in 24-well plates using Lipofectamine 2000 (Invitrogen) according to the manufacturer’s instructions. After 48 h of transfection, luciferase activity was measured using a Dual Luciferase Reporter Assay system (Promega) according to the manufacturer’s instruction. Firefly luciferase activity was normalized to Renilla luciferase activity.

### RNA isolation and qRT-PCR analysis

Total RNA was isolated with RNeasy mini kit (Qiagen, Hamburg, Germany) according to the manufacturer’s instructions. Single-stranded complementary DNA (cDNA) was generated using PrimeScript™ RT Master Mix (Takara, Shiga, Japan) following the manufacturer’s directions. Quantitative RT-PCR was performed in LightCycler 96 (Roche, Penzberg, Germany) using LightCycler FastStart DNA Master SYBR Green I (Roche) and each validated primer. Validated qRT-PCR primers of PAK2 (P287019), Cyclin D3 (P235255), and HPRT1 (P160523) were from Bioneer. For miRNA quantification, small RNA was isolated with *mir*Vana miRNA Isolation Kit (Ambion, Carlsbad, CA) according to the manufacturer’s instructions. Quantitative RT-PCR for miRNA was performed using a TaqMan MicroRNA assay kit (Applied Biosystems, Foster City, CA) and specific primer sets for U6 snRNA (Assay ID: 001973) and mature miR-4779 (Assay ID: 462689_mat) (Applied Biosystems) according to the manufacturer’s instructions. We used HPRT1 and U6 snRNA as internal normalizers for mRNA and miRNA, respectively.

### Western blotting

Total cell lysates were prepared using RIPA buffer (25 mM Tris, pH 7.4, 150 mM NaCl, 1% (v/v) NP-40, 1% (w/v) sodium deoxycholate, 0.1% (w/v) SDS). Each protein sample (20–30 μg) was separated by 10–15% SDS–PAGE and then transferred to nitrocellulose membranes (Millipore, Bedford, MA). Antibodies against PAK2, Cyclin D3, CDK2, CDK4, CDK6, Cyclin D1, p18INK4C, p21Wafl/Cip1, p27Kip1, p53, Caspase 7, PARP, survivin, XIAP, Livin, Bad, phospho-Bad (S112), phospho-Bad (S136), and GAPDH were purchase from Cell Signaling Technology (Beverly, MA). Anti-Mcl-1, anti-Bax, HRP-conjugated goat anti-mouse IgG, and HRP-conjugated goat anti-rabbit IgG were purchased from Santa Cruz Biotechnology (Santa Cruz, CA). Anti-β-actin was purchased from Sigma. The labeled proteins were visualized with Immobilon Western Chemiluminesent HRP Substrate kit (Millipore) or Power Opti-ECL Western blotting Detection reagent (Bionote, Hwaseong, Korea) and the images were captured by ImageQuant LAS 4000 (GE healthcare, Buckinghamshire, UK). The protein band intensity on western blots was quantified and normalized to that of β-actin by the densitometry analysis using ImageJ software (http://rsb.info.nih.gov/ij/).

### Tumor xenograft experiments

Female BALB/c nu/nu mice (4-weeks-old females) were purchased from Central Lab. Animal Inc. (Seoul, Korea) and kept in tight cages with standard rodent chow. A total of 5 × 10^6^ HCT116 cells in 100 µl PBS were subcutaneously injected into the flanks of 5-week old mice. When solid tumors were established (the average tumor volume by 100 mm^3^, 6 days after cell implantation), in vivo-jetPEI/miRNA complex (miR-NC or miR-4779) were treated. Treatment with in vivo-jetPEI/miRNA complexes was done by intratumoral injection with 3 times at 3 day intervals. In vivo-jetPEI (Polyplus Transfection, Illkirch, France) was used as a delivery agent (miRNA 10 μg and in vivo-jetPEI reagent 1.2 µl in 100 µl of 5% glucose for one injection). For ex vivo xenografts, HCT116 cells were transfected with miR-NC or miR-4779 using Lipofectamin RNAiMAX reagent by reverse transfection. After 20 h transfection, miR-NC or miR-4779 transfected HCT116 cells (2 × 10^6^ cells/100 µl PBS) were injected into the both flanks of 5-week old mice. The tumor size was measured by a caliper every 2–3 days from day 5 to day 21 after cell implantation. The volume was calculated by a formula: V = 0.5 A × B^2^, where A is the long diameter and B is the short diameter. The animals were killed on day 22. The tumor was removed from the body and weighed.

### Patient specimens

Collection and use of colon cancer patient specimens were approved by the Institutional Review Board of Korea Institute of Radiological and Medical Sciences (KIRAMS, Seoul, Korea, IRB No. K-1602-002-050). Ten cases of colon cancer specimens and adjacent normal colon tissues were provided by the Radiation Tissue Resources Bank of Korea Cancer Center Hospital (2016-1-01-C/P10) (KCCH, KIRAMS). Samples were frozen and stored in liquid nitrogen until use.

### Statistics

Summary statistics are presented as the mean ± S.D. Where appropriate, data were evaluated by performing a simple comparison between two values using Student’s *t*-test. A *P*-value of <0.05 was considered statistically significant.

## Electronic supplementary material

Supplementary Information

Supplementary Data

Supplementary Table
