# Supplementary material for: MicroRNA miR-4779 suppresses tumor growth by inducing apoptosis and cell cycle arrest through direct targeting of PAK2 and CCND3
Source: Cell Death Dis. 2018 Jan 23;9(2):77. doi: 10.1038/s41419-017-0100-x (PMC5833427; doi:10.1038/s41419-017-0100-x)
Supplement: Supplementary file 1 — Supplementary Information [file 41419_2017_100_MOESM1_ESM.docx]

**[Supplemental Experimental Procedures]**

**Cell culture.** Human colon cancer cell lines HCT116 and HT-29, human lung cancer cell lines A549 and H460, and human breast cancer cell line MCF-7 were obtained from the American Type Culture Collection (ATCC, Rockville, MD, USA). Human normal lung epithelial cell line BEAS-2B and human normal colon fibroblast cell line CCD-18Co was obtained from the ATCC. HCT116, A549, H460, and BEAS-2B cells were maintained in RPMI1640 media (Welgene, Daegu, Korea) supplemented with 10% (v/v) fetal bovine serum (FBS) (Hyclone, Rockford, IL) and 1% (v/v) penicillin and streptomycin (Invitrogen, San Diego, CA). HT29 and MCF-7 cells were maintained in DMEM media (Welgene) supplemented with 10% (v/v) FBS and 1% (v/v) penicillin and streptomycin. CCD-18Co cells were maintained in MEM media (Welgene) supplemented with 10% (v/v) FBS and 1% (v/v) penicillin and streptomycin.

**MTS assay.** We seeded cells into 96-well plates and transfected them with each of the miRNAs. Cell viability was measured after 72 hr with the CellTiter96 Aqueous Non-radioactive Assay (Promega, Madison, WI) as described in the manufacturer’s instruction. Briefly, 20 μl of the MTS solution was added to each well, and the plates were incubated for another 2 hr. The absorbance at 490 nm was measured by microplate reader (Multiskan EX, Thermo LabSystems, Champaign, IL).

**RNA oligoribonucleotides and transfection.** Synthetic miRNA mimics were prepared by Genolution as RNA duplexes designed from the sequence of miR-4779 (5’- UAGGAGGGAAUAGUAAAAGCAG-3’) and miR-NC (negative control with scrambled sequence) (5’- ACUCUAUCUGCACGCUGACUU-3’). Validated small interfering RNA (si-RNA) duplex of human PAK2 (si-PAK2, 100266) and the negative control with scrambled sequence (si-NC, SN1003) were purchased from Bioneer. For RNA interference, cells were transfected with 20 nM si-RNAs and 50 nM miRNA mimics using Lipofectamin RNAiMAX reagent (invitrogen) by reverse transfection according to the manufacturer’s protocol.

**RNA isolation and qRT-PCR analysis.** For miRNA quantification, small RNA was isolated with *mir*Vana miRNA Isolation Kit (Ambion, Carlsbad, CA) according to the manufacturer’s instructions. Quantitative RT-PCR for miRNA was performed using a TaqMan MicroRNA assay kit (Applied Biosystems, Foster City, CA) and specific primer sets for U6 snRNA (Assay ID: 001973) and mature miR-4779 (Assay ID: 462689_mat) (Applied Biosystems) according to the manufacturer’s instructions. We used U6 snRNA as internal normalizers for miRNA.

**Western blotting.** Total cell lysates were prepared using RIPA buffer (25mM Tris, pH 7.4, 150 mM NaCl, 1% (v/v) NP-40, 1% (w/v) sodium deoxycholate, 0.1% (w/v) SDS). Each protein sample (20~30 μg) was separated by 10-15% SDS-PAGE and then transferred to nitrocellulose membranes (Millipore, Bedford, MA). Antibodies against PAK2, Cyclin D3, Bad, phospho-Bad (S112), and phospho-Bad (S136) were purchase from Cell Signaling Technology (Beverly, MA). HRP-conjugated goat anti-mouse IgG and HRP-conjugated goat anti-rabbit IgG were purchased from Santa Cruz Biotechnology (Santa Cruz, CA). Anti-β-actin was purchased from Sigma (St. Louis, MO). The labeled proteins were visualized with Immobilon Western Chemiluminesent HRP Substrate kit (Millipore) or Power Opti-ECL Western blotting Detection reagent (Bionote, Hwaseong, Korea) and the images were captured by ImageQuant LAS 4000 (GE healthcare, Buckinghamshire, UK).

**[Supplemental Figure Legends]**

**Supplementary Figure 1. Effect of 30 miRNAs on the cell viability in HCT116 cells.** HCT116 cells were transfected with miR-NC (negative control) and 30 miRNAs (#1-30) for 72 hr and cell viability was determined by MTS assay. The non-transfected control (cont) was normalized to 100% cell viability and the effects of the miR-NC and 30 miRNAs were calculated accordingly. Data are presented as averages of triplicate measurements with error bars representing standard deviations. #20 (blue bar) indicates miR-4779.

**Supplementary Figure 2. Effect of 30 miRNAs on the cell viability in various cancer cells.** A549, H460, MCF7, and HT-29 cells were transfected with miR-NC and 30 miRNAs (#1-30) for 72 hr and cell viability was determined by MTS assay. #20 (blue bar) indicates miR-4779.

**Supplementary Figure 3. Effects of miR-4779 on the cell viability and PARP cleavage in various cancer cells. (A-B)** HCT116, A549, H460, MCF7, and HT-29 cells were transfected with miR-NC or miR-4779 for 48 hr. Cell viability was observed by microscopy (**A**). Cell images were taken using phase contrast microscopy. The protein levels of cleaved PARP (lower band, 87 kDa) were determined by western blot analysis using the corresponding antibody (**B**). β-actin was used as a loading control.

**Supplementary Figure 4. Sequence alignment of 3′UTR of PAK2 (A) and CCND3 (B) binding sites for miR-4779 in 18 different species.** The aligned sequences of two 3′UTR of PAK2 (894-900 and 2106-2113) and one 3′UTR of CCND3 (795-801) (red) complementary to the seed sequence of the miR-4779 are shown (left). The red boxes show the species of conserved (right).

**Supplementary Figure 5. Effect of miR-4779 inhibitor on the apoptosis, colony formation, and anchorage-independent growth.** HCT116 cells were transfected with mimics or inhibitors of miR-NC and miR-4779. At 48 hr after transfection, apoptosis was measured by flow cytometric analysis of cells stained with Annexin V-FITC and PI (A). Right, the quantified apoptotic cell population was estimated. At 24 hr after transfection, cells were re-plated and performed colony formation (B) and soft agar assays (C). All data are presented as averages of triplicate measurements with error bars representing standard deviations. **P<0.01 and ***P<0.001.

**Supplementary Figure 6. Effect of mimicking of miR-4779 or knock-down of PAK2 on the Bad phosphorylation.** HCT116 cells were transfected with miR-NC, miR-4779, si-NC, and si-PAK2 for 48 hr, and the protein level of Bad, p-Bad (S112), p-Bad (S136) were determined by western blot analysis using the corresponding antibodies. β-actin was used as a loading control.

**Supplementary Figure 7. Overall survival of miR-4779 in esophageal squamous cell carcinoma patients**. Kaplan-Meier analysis of esophageal squamous cell carcinoma patients was available in the MIRUMIR (probe ID: hsa-miR-4779). P value is 0.6

**Supplementary Figure 8. Correlation analysis of miR-4779 expression and PAK2 (A) or CCND3 (B) expression.** The relative miR-4779 expression levels (Fig. 6A) and the relative PAK2 or CCND3 protein levels (Fig. 6B) from colon cancer patients (20 samples, normal and tumor) were analyzed by Pearson correlation and linear regression using Graphpad Prism 5.

**Supplementary Figure 9. Comparison of expression of miR-4779 and target genes in various cancer cells and normal cells. (A)** Comparison of miR-4779 expression in HCT116, A549, H460, MCF7, and HT29 cells. Small RNA was extracted from each cell lysates and qRT-PCR was performed. U6 RNA was used as a normalizer. The expression level of miR-4779 in HCT116 cells was set at 1, and the relative amounts of miR-4779 at the other cancer cells were plotted as fold induction. Error bars represent mean ± SEM. **(B)** Comparison of protein expression of PAK2 and CCND3 in HCT116, A549, H460, MCF7, and HT29 cells. Equal amount of each cell lysates were subjected to western blot analysis using the corresponding antibodies. β-actin was used as a loading control. **(C)** Comparison of miR-4779 expression in HCT116, CCD-18Co, A549, and BEAS-2B cells. (D) Comparison of protein expression of PAK2 and CCND3 in HCT116, CCD-18Co, A549, and BEAS-2B cells.
