# Supplementary material for: MicroRNA miR-4779 suppresses tumor growth by inducing apoptosis and cell cycle arrest through direct targeting of PAK2 and CCND3
Source: Cell Death Dis. 2018 Jan 23;9(2):77. doi: 10.1038/s41419-017-0100-x (PMC5833427; doi:10.1038/s41419-017-0100-x)
Supplement: Supplementary file 2 — Supplementary Data [file 41419_2017_100_MOESM2_ESM.pptx]

## Slide 1
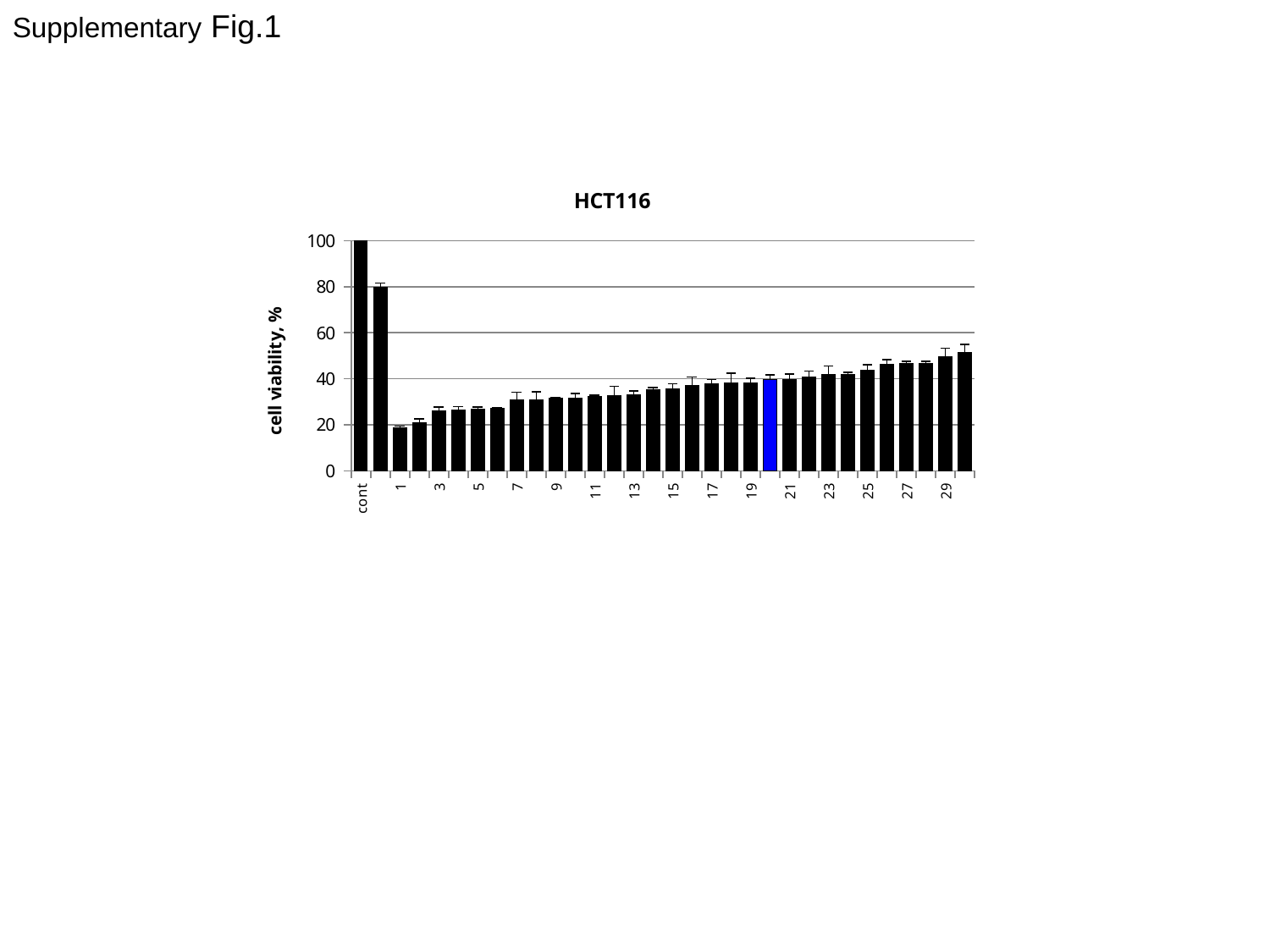

Supplementary Fig.1
### Chart: HCT116
| Category | |
|---|---|
| cont | 100.0 |
| miR-NC | 79.65 |
| 1 | 18.7 |
| 2 | 20.966666666666665 |
| 3 | 26.2 |
| 4 | 26.599999999999998 |
| 5 | 26.833333333333332 |
| 6 | 27.066666666666666 |
| 7 | 30.866666666666664 |
| 8 | 31.0 |
| 9 | 31.46666666666667 |
| 10 | 31.600000000000005 |
| 11 | 32.199999999999996 |
| 12 | 32.833333333333336 |
| 13 | 33.0 |
| 14 | 35.1 |
| 15 | 35.43333333333333 |
| 16 | 37.1 |
| 17 | 37.96666666666667 |
| 18 | 38.1 |
| 19 | 38.26666666666666 |
| 20 | 39.699999999999996 |
| 21 | 39.833333333333336 |
| 22 | 40.766666666666666 |
| 23 | 41.800000000000004 |
| 24 | 41.86666666666667 |
| 25 | 43.699999999999996 |
| 26 | 46.26666666666667 |
| 27 | 46.63333333333333 |
| 28 | 46.699999999999996 |
| 29 | 49.43333333333334 |
| 30 | 51.26666666666667 |

## Slide 2
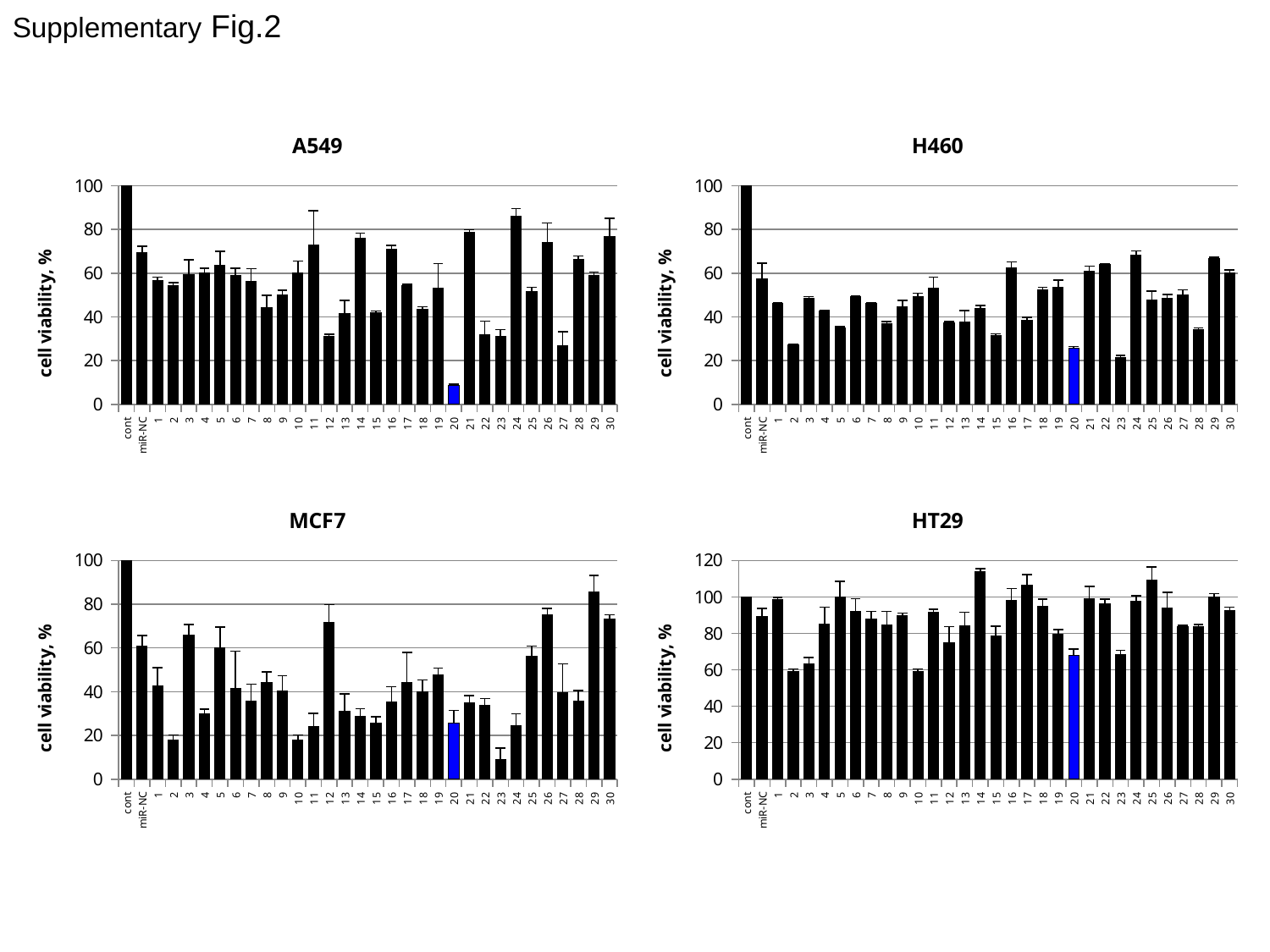

Supplementary Fig.2
### Chart: A549
| Category | |
|---|---|
| cont | 100.0 |
| miR-NC | 69.5 |
| 1 | 56.6 |
| 2 | 54.25 |
| 3 | 59.449999999999996 |
| 4 | 60.3 |
| 5 | 63.45 |
| 6 | 58.95 |
| 7 | 56.349999999999994 |
| 8 | 44.4 |
| 9 | 50.0 |
| 10 | 60.25 |
| 11 | 72.85 |
| 12 | 31.15 |
| 13 | 41.5 |
| 14 | 76.0 |
| 15 | 42.0 |
| 16 | 71.05000000000001 |
| 17 | 54.35 |
| 18 | 43.400000000000006 |
| 19 | 53.0 |
| 20 | 8.7 |
| 21 | 78.55 |
| 22 | 32.0 |
| 23 | 31.2 |
| 24 | 85.85 |
| 25 | 51.6 |
| 26 | 73.94999999999999 |
| 27 | 26.950000000000003 |
| 28 | 66.19999999999999 |
| 29 | 59.05 |
| 30 | 76.8 |
### Chart: H460
| Category | |
|---|---|
| cont | 100.0 |
| miR-NC | 57.4 |
| 1 | 46.2 |
| 2 | 27.3 |
| 3 | 48.5 |
| 4 | 42.8 |
| 5 | 34.8 |
| 6 | 49.1 |
| 7 | 46.1 |
| 8 | 36.7 |
| 9 | 44.7 |
| 10 | 49.3 |
| 11 | 53.2 |
| 12 | 37.3 |
| 13 | 37.6 |
| 14 | 43.9 |
| 15 | 31.3 |
| 16 | 62.5 |
| 17 | 38.6 |
| 18 | 52.4 |
| 19 | 53.4 |
| 20 | 25.5 |
| 21 | 60.9 |
| 22 | 63.8 |
| 23 | 21.3 |
| 24 | 68.4 |
| 25 | 47.6 |
| 26 | 48.4 |
| 27 | 50.1 |
| 28 | 34.1 |
| 29 | 66.5 |
| 30 | 60.3 |
### Chart: MCF7
| Category | |
|---|---|
| cont | 100.0 |
| miR-NC | 60.849999999999994 |
| 1 | 42.75 |
| 2 | 17.950000000000003 |
| 3 | 65.95 |
| 4 | 29.8 |
| 5 | 59.900000000000006 |
| 6 | 41.5 |
| 7 | 35.55 |
| 8 | 44.25 |
| 9 | 40.25 |
| 10 | 17.950000000000003 |
| 11 | 24.0 |
| 12 | 71.7 |
| 13 | 30.950000000000003 |
| 14 | 28.9 |
| 15 | 25.450000000000003 |
| 16 | 35.400000000000006 |
| 17 | 44.2 |
| 18 | 39.849999999999994 |
| 19 | 47.7 |
| 20 | 25.65 |
| 21 | 34.900000000000006 |
| 22 | 33.8 |
| 23 | 9.15 |
| 24 | 24.35 |
| 25 | 56.15 |
| 26 | 75.05000000000001 |
| 27 | 39.599999999999994 |
| 28 | 35.55 |
| 29 | 85.65 |
| 30 | 73.4 |
### Chart: HT29
| Category | |
|---|---|
| cont | 100.0 |
| miR-NC | 89.4 |
| 1 | 98.35 |
| 2 | 59.1 |
| 3 | 63.150000000000006 |
| 4 | 85.25 |
| 5 | 100.0 |
| 6 | 92.2 |
| 7 | 87.7 |
| 8 | 84.55 |
| 9 | 89.5 |
| 10 | 59.1 |
| 11 | 91.65 |
| 12 | 75.05 |
| 13 | 84.2 |
| 14 | 113.9 |
| 15 | 78.45 |
| 16 | 98.0 |
| 17 | 106.55 |
| 18 | 94.69999999999999 |
| 19 | 79.44999999999999 |
| 20 | 68.05000000000001 |
| 21 | 99.0 |
| 22 | 96.3 |
| 23 | 68.6 |
| 24 | 97.45 |
| 25 | 109.19999999999999 |
| 26 | 94.05 |
| 27 | 83.55 |
| 28 | 83.55000000000001 |
| 29 | 100.05 |
| 30 | 92.65 |

## Slide 3
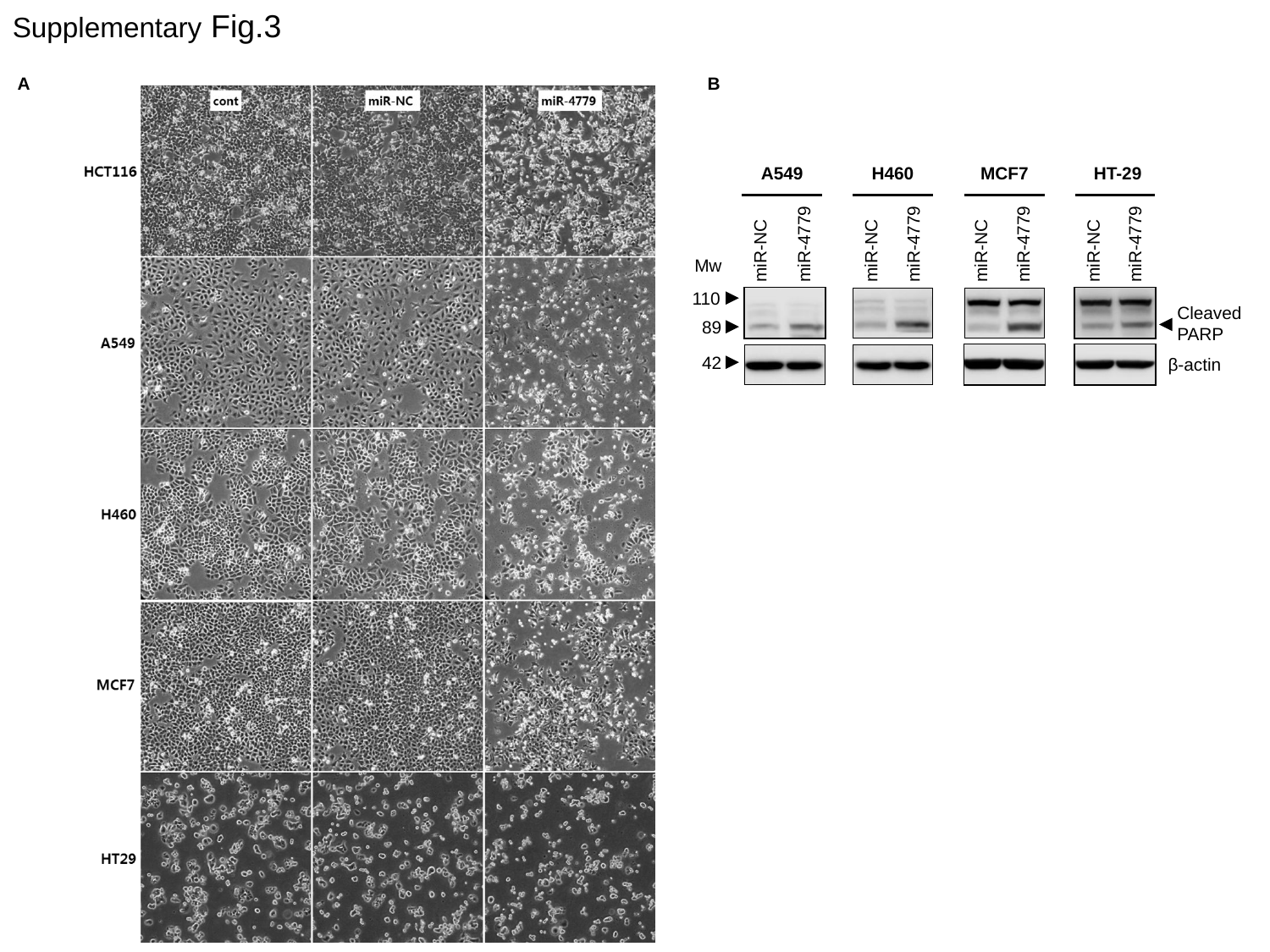

Supplementary Fig.3
A
B
MCF7
A549
H460
HT-29
miR-4779
miR-4779
miR-4779
miR-4779
miR-NC
miR-NC
miR-NC
miR-NC
 Mw
110
Cleaved PARP
 89
β-actin
 42

## Slide 4
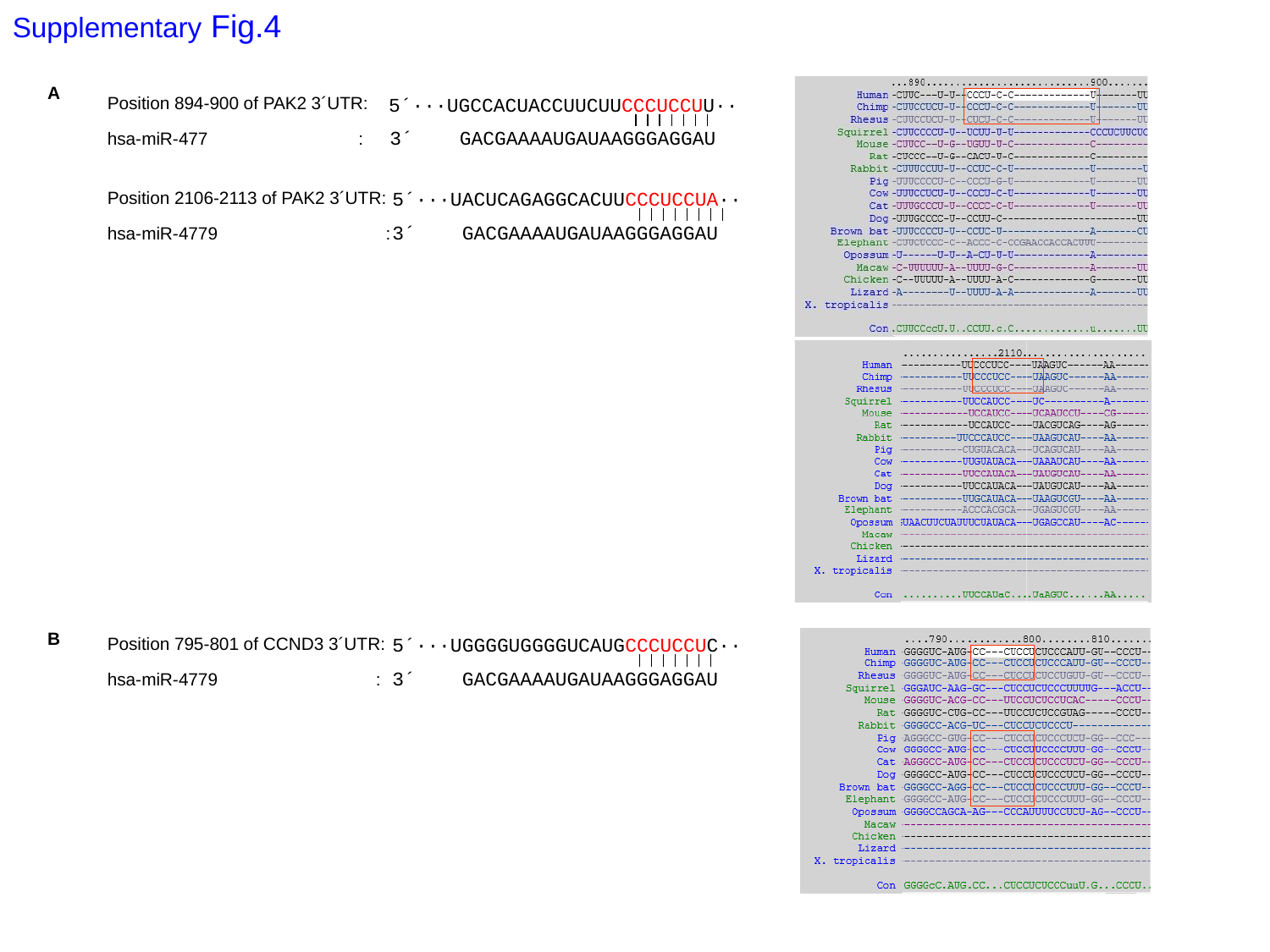

Supplementary Fig.4
A
Position 894-900 of PAK2 3´UTR:
5´···UGCCACUACCUUCUUCCCUCCUU··
3´ GACGAAAAUGAUAAGGGAGGAU
hsa-miR-477 :
Position 2106-2113 of PAK2 3´UTR:
5´···UACUCAGAGGCACUUCCCUCCUA··
3´ GACGAAAAUGAUAAGGGAGGAU
hsa-miR-4779		 :
B
Position 795-801 of CCND3 3´UTR:
5´···UGGGGUGGGGUCAUGCCCUCCUC··
3´ GACGAAAAUGAUAAGGGAGGAU
hsa-miR-4779		 :

## Slide 5
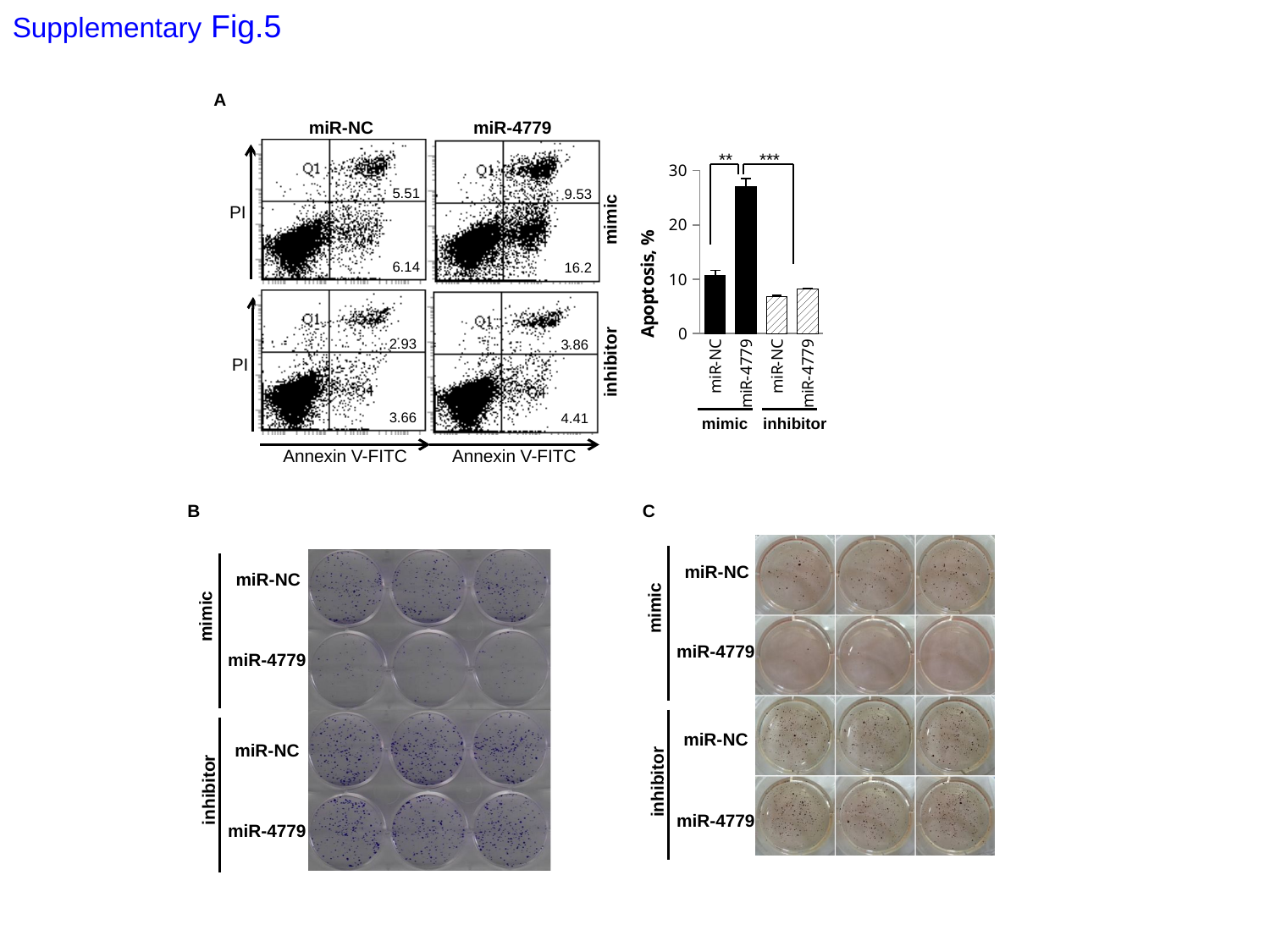

Supplementary Fig.5
A
miR-NC
miR-4779
5.51
6.14
9.53
16.2
PI
mimic
2.93
3.66
3.86
4.41
PI
inhibitor
Annexin V-FITC
Annexin V-FITC
**
***
### Chart
| Category | |
|---|---|
| miR-NC | 10.75 |
| miR-4779 | 27.1 |
| miR-NC | 6.8 |
| miR-4779 | 8.15 |mimic
inhibitor
B
C
miR-NC
mimic
miR-4779
miR-NC
inhibitor
miR-4779
miR-NC
mimic
miR-4779
miR-NC
inhibitor
miR-4779

## Slide 6
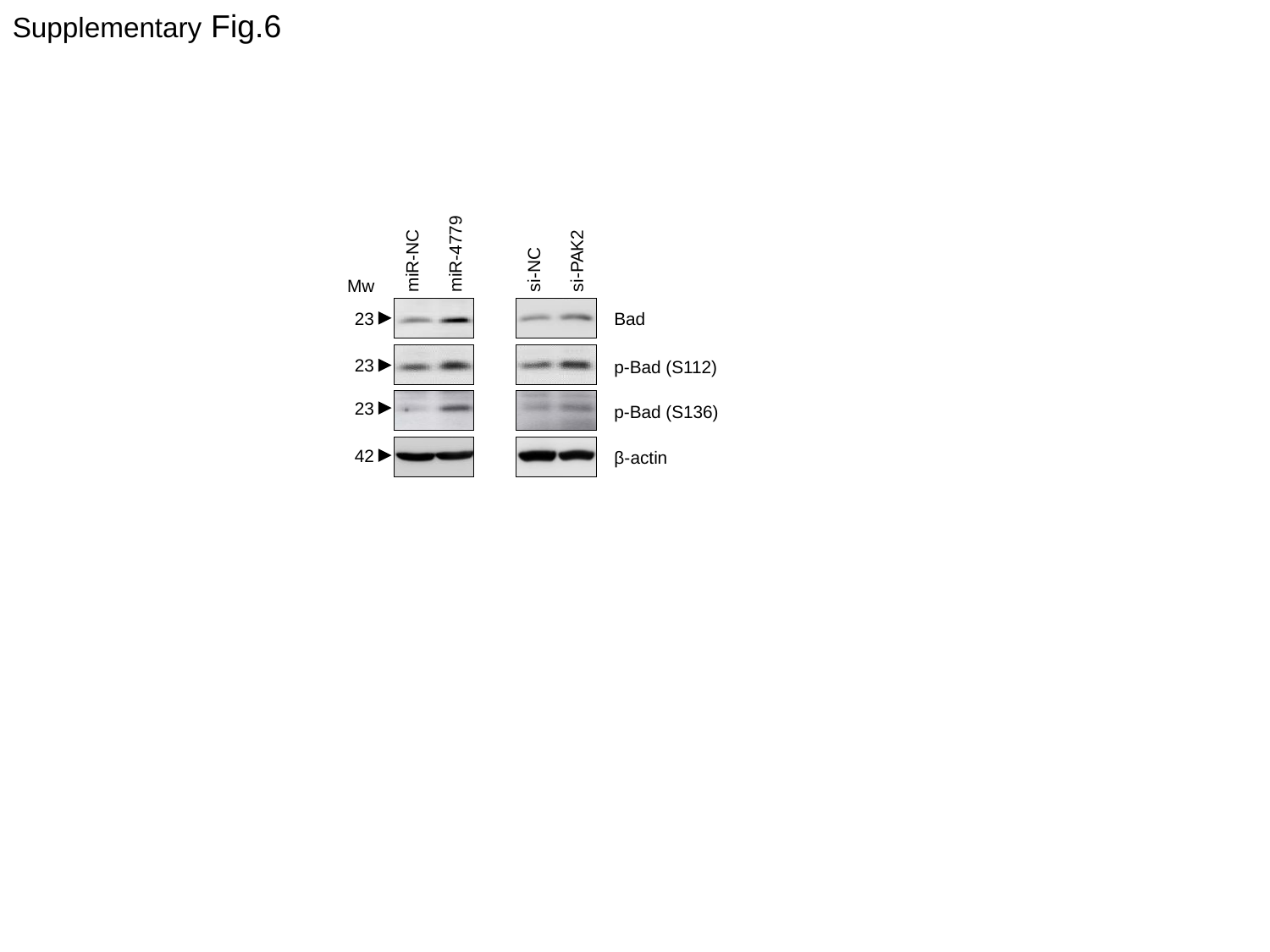

Supplementary Fig.6
miR-4779
miR-NC
si-PAK2
si-NC
 Mw
Bad
 23
p-Bad (S112)
 23
p-Bad (S136)
 23
β-actin
 42

## Slide 7
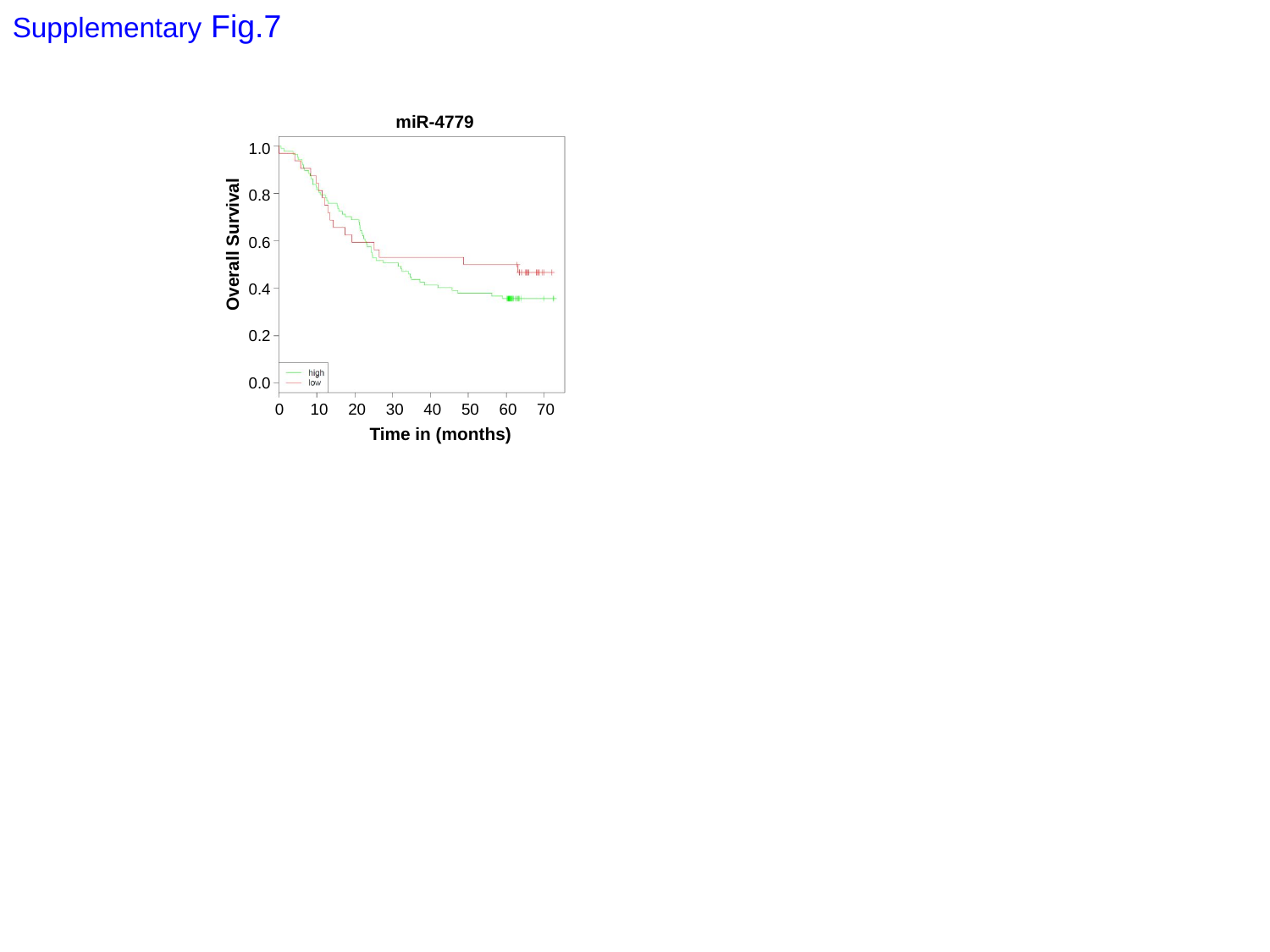

Supplementary Fig.7
miR-4779
1.0
0.8
0.6
Overall Survival
0.4
0.2
0.0
0
10
20
30
40
50
60
70
Time in (months)

## Slide 8
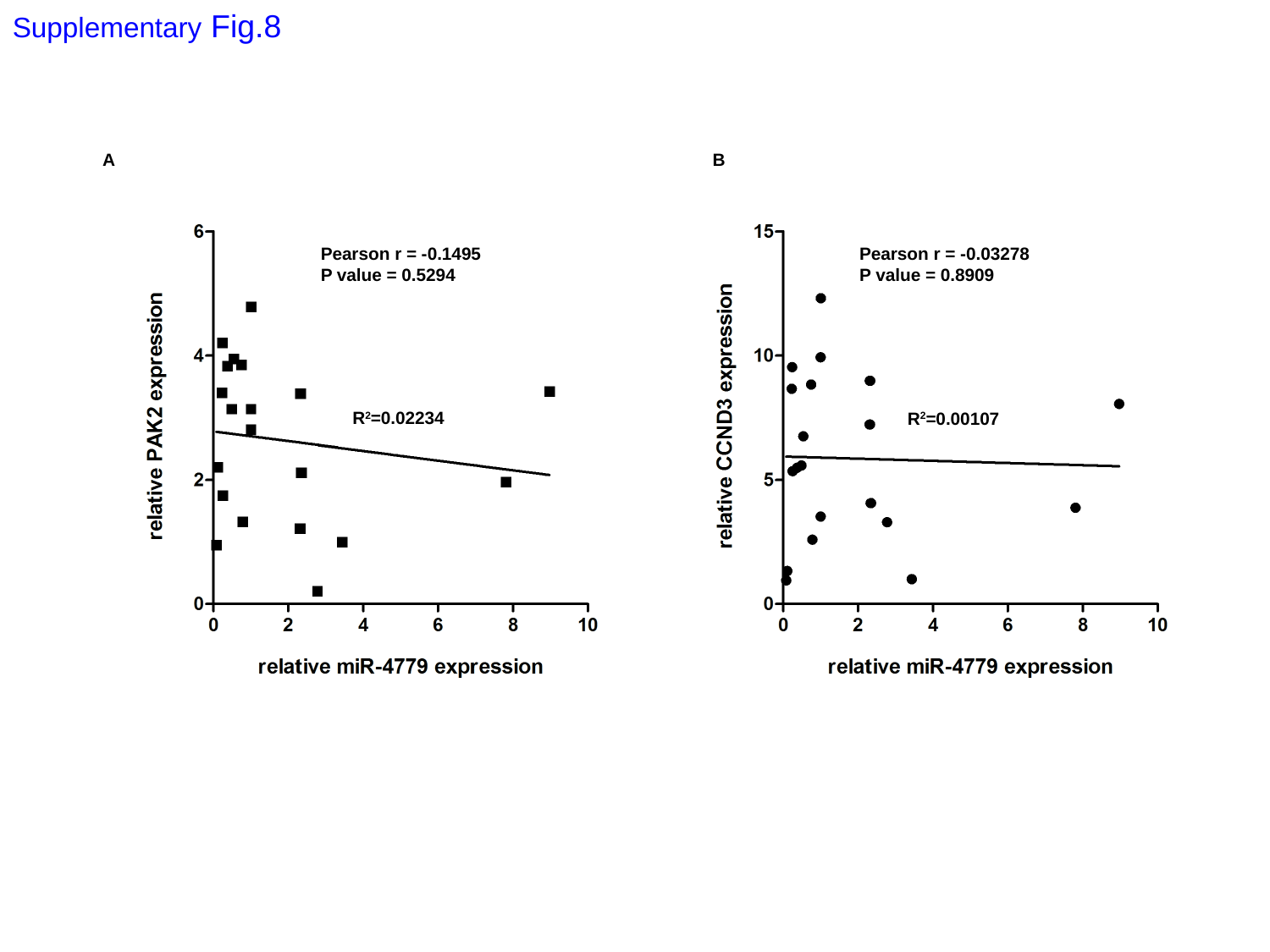

Supplementary Fig.8
A
B
Pearson r = -0.1495
P value = 0.5294
Pearson r = -0.03278
P value = 0.8909
R2=0.02234
R2=0.00107

## Slide 9
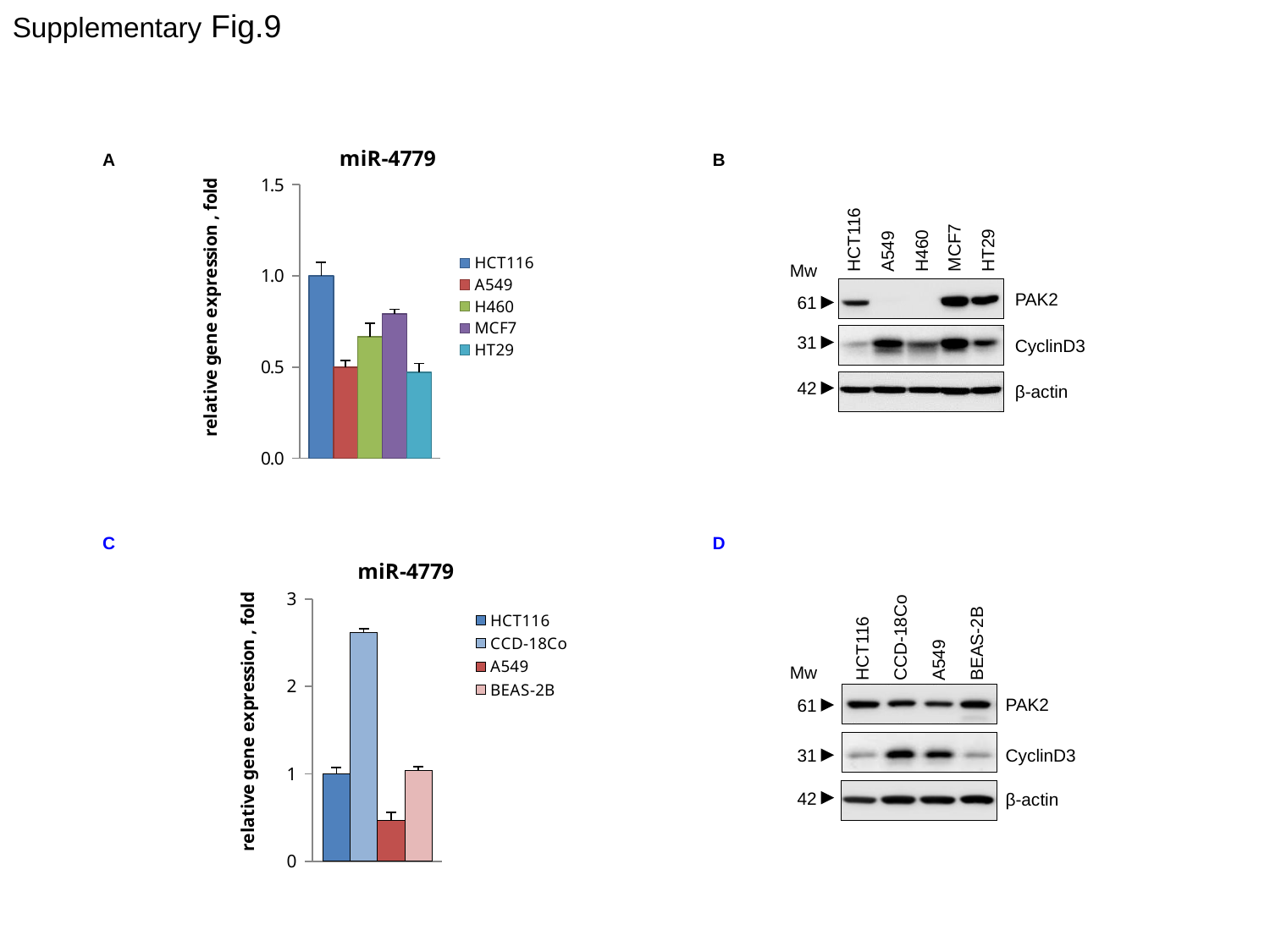

Supplementary Fig.9
### Chart: miR-4779
| Category | HCT116 | A549 | H460 | MCF7 | HT29 |
|---|---|---|---|---|---|A
B
HCT116
A549
MCF7
HT29
H460
 Mw
PAK2
 61
CyclinD3
 31
β-actin
 42
C
D
### Chart: miR-4779
| Category | HCT116 | CCD-18Co | A549 | BEAS-2B |
|---|---|---|---|---|CCD-18Co
HCT116
BEAS-2B
A549
 Mw
PAK2
 61
CyclinD3
 31
β-actin
 42
