# Supplementary material for: MicroRNA miR-4779 suppresses tumor growth by inducing apoptosis and cell cycle arrest through direct targeting of PAK2 and CCND3
Source: Cell Death Dis. 2018 Jan 23;9(2):77. doi: 10.1038/s41419-017-0100-x (PMC5833427; doi:10.1038/s41419-017-0100-x)
Supplement: Supplementary file 3 — Supplementary Table [file 41419_2017_100_MOESM3_ESM.docx]

**Supplementary Table 1** List of miRNA librariesused for screening

| # | **mature miRNA mimic** | **accession #** | **#** | **mature miRNA mimic** | **accession** |
| --- | --- | --- | --- | --- | --- |
| 1 | has-let-7f | MIMAT0000067 | 43 | hsa-miR-1304-3p | MIMAT0022720 |
| 2 | hsa-let-7a | MIMAT0000062 | 44 | hsa-miR-1306-5p | MIMAT0022726 |
| 3 | hsa-let-7b | MIMAT0000063 | 45 | hsa-miR-1307-5p | MIMAT0022727 |
| 4 | hsa-let-7d | MIMAT0000065 | 46 | hsa-miR-130a | MIMAT0000425 |
| 5 | hsa-let-7i* | MIMAT0004585 | 47 | hsa-miR-130b | MIMAT0000691 |
| 6 | hsa-miR-1 | MIMAT0000416 | 48 | hsa-miR-132 | MIMAT0000426 |
| 7 | hsa-miR-100 | MIMAT0000098 | 49 | hsa-miR-133a | MIMAT0000427 |
| 8 | hsa-miR-101 | MIMAT0000099 | 50 | hsa-miR-133b | MIMAT0000770 |
| 9 | hsa-miR-103a | MIMAT0000101 | 51 | hsa-miR-134 | MIMAT0000447 |
| 10 | hsa-miR-103b | MIMAT0007402 | 52 | hsa-miR-1343 | MIMAT0019776 |
| 11 | hsa-miR-105 | MIMAT0000102 | 53 | hsa-miR-135a | MIMAT0000428 |
| 12 | hsa-miR-106a | MIMAT0000103 | 54 | hsa-miR-135b | MIMAT0000758 |
| 13 | hsa-miR-106b | MIMAT0000680 | 55 | hsa-miR-136 | MIMAT0000448 |
| 14 | hsa-miR-107 | MIMAT0000104 | 56 | hsa-miR-137 | MIMAT0000429 |
| 15 | hsa-miR-10a | MIMAT0000253 | 57 | hsa-miR-138 | MIMAT0000430 |
| 16 | hsa-miR-10b | MIMAT0000254 | 58 | hsa-miR-140-5p | MIMAT0000431 |
| 17 | hsa-miR-1181 | MIMAT0005826 | 59 | hsa-miR-141 | MIMAT0000432 |
| 18 | hsa-miR-1185 | MIMAT0005798 | 60 | hsa-miR-142-3p | MIMAT0000434 |
| 19 | hsa-miR-1185-1-3p | MIMAT0022838 | 61 | hsa-miR-142-5p | MIMAT0000433 |
| 20 | hsa-miR-1185-2-3p | MIMAT0022713 | 62 | hsa-miR-143 | MIMAT0000435 |
| 21 | hsa-miR-1197 | MIMAT0005955 | 63 | hsa-miR-144 | MIMAT0000436 |
| 22 | hsa-miR-122 | MIMAT0000421 | 64 | hsa-miR-144* | MIMAT0004600 |
| 23 | hsa-miR-1226* | MIMAT0005576 | 65 | hsa-miR-145 | MIMAT0000437 |
| 24 | hsa-miR-124 | MIMAT0000422 | 66 | hsa-miR-146a | MIMAT0000449 |
| 25 | hsa-miR-1244 | MIMAT0005896 | 67 | hsa-miR-146b-5p | MIMAT0002809 |
| 26 | hsa-miR-1247-3p | MIMAT0022721 | 68 | hsa-miR-148b | MIMAT0000759 |
| 27 | hsa-miR-1255b-2-3p | MIMAT0022725 | 69 | hsa-miR-149 | MIMAT0000450 |
| 28 | hsa-miR-125a-5p | MIMAT0000443 | 70 | hsa-miR-150 | MIMAT0000451 |
| 29 | hsa-miR-125b | MIMAT0000423 | 71 | hsa-miR-151-5p | MIMAT0004697 |
| 30 | hsa-miR-126 | MIMAT0000445 | 72 | hsa-miR-152 | MIMAT0000438 |
| 31 | hsa-miR-1260b | MIMAT0015041 | 73 | hsa-miR-153 | MIMAT0000439 |
| 32 | hsa-miR-1271-3p | MIMAT0022712 | 74 | hsa-miR-154 | MIMAT0000452 |
| 33 | hsa-miR-1273g-3p | MIMAT0022742 | 75 | hsa-miR-155 | MIMAT0000646 |
| 34 | hsa-miR-127-5p | MIMAT0004604 | 76 | hsa-miR-155* | MIMAT0004658 |
| 35 | hsa-miR-1277-5p | MIMAT0022724 | 77 | hsa-miR-15a | MIMAT0000068 |
| 36 | hsa-miR-128 | MIMAT0000424 | 78 | hsa-miR-16 | MIMAT0000069 |
| 37 | hsa-miR-1283 | MIMAT0005799 | 79 | hsa-miR-17 | MIMAT0000070 |
| 38 | hsa-miR-1285 | MIMAT0005876 | 80 | hsa-miR-17* | MIMAT0000071 |
| 39 | hsa-miR-1285-5p | MIMAT0022719 | 81 | hsa-miR-181a | MIMAT0000256 |
| 40 | hsa-miR-1295b-3p | MIMAT0022294 | 82 | hsa-miR-181b | MIMAT0000257 |
| 41 | hsa-miR-1295b-5p | MIMAT0022293 | 83 | hsa-miR-181b-3p | MIMAT0022692 |
| 42 | hsa-miR-129-5p | MIMAT0000242 | 84 | hsa-miR-181c | MIMAT0000258 |

**Supplementary Table 1** *Continued*

| # | **mature miRNA mimic** | **accession #** | **#** | **mature miRNA mimic** | **accession** |
| --- | --- | --- | --- | --- | --- |
| 85 | hsa-miR-181d | MIMAT0002821 | 127 | hsa-miR-218 | MIMAT0000275 |
| 86 | hsa-miR-182 | MIMAT0000259 | 128 | hsa-miR-219-1-3p | MIMAT0004567 |
| 87 | hsa-miR-183 | MIMAT0000261 | 129 | hsa-miR-219-5p | MIMAT0000276 |
| 88 | hsa-miR-184 | MIMAT0000454 | 130 | hsa-miR-22 | MIMAT0000077 |
| 89 | hsa-miR-185 | MIMAT0000455 | 131 | hsa-miR-221 | MIMAT0000278 |
| 90 | hsa-miR-188-5p | MIMAT0000457 | 132 | hsa-miR-221* | MIMAT0004568 |
| 91 | hsa-miR-18a | MIMAT0000072 | 133 | hsa-miR-222 | MIMAT0000279 |
| 92 | hsa-miR-18b | MIMAT0001412 | 134 | hsa-miR-222* | MIMAT0004569 |
| 93 | hsa-miR-1908 | MIMAT0007881 | 135 | hsa-miR-223 | MIMAT0000280 |
| 94 | hsa-miR-1915 | MIMAT0007892 | 136 | hsa-miR-224 | MIMAT0000281 |
| 95 | hsa-miR-192 | MIMAT0000222 | 137 | hsa-miR-23a | MIMAT0000078 |
| 96 | hsa-miR-193a-5p | MIMAT0004614 | 138 | hsa-miR-23a* | MIMAT0004496 |
| 97 | hsa-miR-193b | MIMAT0002819 | 139 | hsa-miR-23b | MIMAT0000418 |
| 98 | hsa-miR-194 | MIMAT0000460 | 140 | hsa-miR-24 | MIMAT0000080 |
| 99 | hsa-miR-195 | MIMAT0000461 | 141 | hsa-miR-24-1* | MIMAT0004497 |
| 100 | hsa-miR-196a | MIMAT0000226 | 142 | hsa-miR-25 | MIMAT0000081 |
| 101 | hsa-miR-196b | MIMAT0001080 | 143 | hsa-miR-26a | MIMAT0000082 |
| 102 | hsa-miR-197 | MIMAT0000227 | 144 | hsa-miR-26b | MIMAT0000083 |
| 103 | hsa-miR-197-5p | MIMAT0022691 | 145 | hsa-miR-27a | MIMAT0000084 |
| 104 | hsa-miR-199a-5p | MIMAT0000231 | 146 | hsa-miR-27b | MIMAT0000419 |
| 105 | hsa-miR-19a | MIMAT0000073 | 147 | hsa-miR-27b* | MIMAT0004588 |
| 106 | hsa-miR-200b | MIMAT0000318 | 148 | hsa-miR-28-3p | MIMAT0004502 |
| 107 | hsa-miR-200c | MIMAT0000617 | 149 | hsa-miR-28-5p | MIMAT0000085 |
| 108 | hsa-miR-202 | MIMAT0002811 | 150 | hsa-miR-296-3p | MIMAT0004679 |
| 109 | hsa-miR-203 | MIMAT0000264 | 151 | hsa-miR-296-5p | MIMAT0000690 |
| 110 | hsa-miR-204 | MIMAT0000265 | 152 | hsa-miR-299-3p | MIMAT0000687 |
| 111 | hsa-miR-204-3p | MIMAT0022693 | 153 | hsa-miR-299-5p | MIMAT0002890 |
| 112 | hsa-miR-205 | MIMAT0000266 | 154 | hsa-miR-29b | MIMAT0000100 |
| 113 | hsa-miR-205* | MIMAT0009197 | 155 | hsa-miR-301a | MIMAT0000688 |
| 114 | hsa-miR-206 | MIMAT0000462 | 156 | hsa-miR-301a-5p | MIMAT0022696 |
| 115 | hsa-miR-208b | MIMAT0004960 | 157 | hsa-miR-302a | MIMAT0000684 |
| 116 | hsa-miR-20a | MIMAT0000075 | 158 | hsa-miR-302a* | MIMAT0000683 |
| 117 | hsa-miR-20b | MIMAT0001413 | 159 | hsa-miR-302b | MIMAT0000715 |
| 118 | hsa-miR-21 | MIMAT0000076 | 160 | hsa-miR-302b* | MIMAT0000714 |
| 119 | hsa-miR-21* | MIMAT0004494 | 161 | hsa-miR-302c | MIMAT0000717 |
| 120 | hsa-miR-210 | MIMAT0000267 | 162 | hsa-miR-302d | MIMAT0000718 |
| 121 | hsa-miR-211 | MIMAT0000268 | 163 | hsa-miR-30c | MIMAT0000244 |
| 122 | hsa-miR-211-3p | MIMAT0022694 | 164 | hsa-miR-30e | MIMAT0000692 |
| 123 | hsa-miR-212 | MIMAT0000269 | 165 | hsa-miR-31 | MIMAT0000089 |
| 124 | hsa-miR-212-5p | MIMAT0022695 | 166 | hsa-miR-31* | MIMAT0004504 |
| 125 | hsa-miR-214 | MIMAT0000271 | 167 | hsa-miR-3131 | MIMAT0014996 |
| 126 | hsa-miR-215 | MIMAT0000272 | 168 | hsa-miR-3162 | MIMAT0015036 |

**Supplementary Table 1** *Continued*

| # | **mature miRNA mimic** | **accession #** | **#** | **mature miRNA mimic** | **accession** |
| --- | --- | --- | --- | --- | --- |
| 169 | hsa-miR-3184-3p | MIMAT0022731 | 211 | hsa-miR-379* | MIMAT0004690 |
| 170 | hsa-miR-3190-3p | MIMAT0022839 | 212 | hsa-miR-381 | MIMAT0000736 |
| 171 | hsa-miR-3191-5p | MIMAT0022732 | 213 | hsa-miR-382-3p | MIMAT0022697 |
| 172 | hsa-miR-32 | MIMAT0000090 | 214 | hsa-miR-412 | MIMAT0002170 |
| 173 | hsa-miR-320a | MIMAT0000510 | 215 | hsa-miR-4281 | MIMAT0016907 |
| 174 | hsa-miR-320b | MIMAT0005792 | 216 | hsa-miR-429 | MIMAT0001536 |
| 175 | hsa-miR-320c | MIMAT0005793 | 217 | hsa-miR-433 | MIMAT0001627 |
| 176 | hsa-miR-320d | MIMAT0006764 | 218 | hsa-miR-4433-5p | MIMAT0020956 |
| 177 | hsa-miR-323b-5p | MIMAT0001630 | 219 | hsa-miR-4482-3p | MIMAT0020958 |
| 178 | hsa-miR-325 | MIMAT0000771 | 220 | hsa-miR-449a | MIMAT0001541 |
| 179 | hsa-miR-326 | MIMAT0000756 | 221 | hsa-miR-449b | MIMAT0003327 |
| 180 | hsa-miR-328 | MIMAT0000752 | 222 | hsa-miR-449c | MIMAT0010251 |
| 181 | hsa-miR-329 | MIMAT0001629 | 223 | hsa-miR-450a-3p | MIMAT0022700 |
| 182 | hsa-miR-335 | MIMAT0000765 | 224 | hsa-miR-451 | MIMAT0001631 |
| 183 | hsa-miR-33a | MIMAT0000091 | 225 | hsa-miR-452* | MIMAT0001636 |
| 184 | hsa-miR-33b | MIMAT0003301 | 226 | hsa-miR-4524b-3p | MIMAT0022256 |
| 185 | hsa-miR-340 | MIMAT0004692 | 227 | hsa-miR-4524b-5p | MIMAT0022255 |
| 186 | hsa-miR-342-3p | MIMAT0000753 | 228 | hsa-miR-4536-3p | MIMAT0020959 |
| 187 | hsa-miR-342-5p | MIMAT0004694 | 229 | hsa-miR-454 | MIMAT0003885 |
| 188 | hsa-miR-345 | MIMAT0000772 | 230 | hsa-miR-466 | MIMAT0015002 |
| 189 | hsa-miR-345-3p | MIMAT0022698 | 231 | hsa-miR-4666b | MIMAT0022485 |
| 190 | hsa-miR-346 | MIMAT0000773 | 232 | hsa-miR-4768-3p | MIMAT0019921 |
| 191 | hsa-miR-34a | MIMAT0000255 | 233 | hsa-miR-4768-5p | MIMAT0019920 |
| 192 | hsa-miR-3529-3p | MIMAT0022741 | 234 | hsa-miR-4769-3p | MIMAT0019923 |
| 193 | hsa-miR-3591-5p | MIMAT0019876 | 235 | hsa-miR-4769-5p | MIMAT0019922 |
| 194 | hsa-miR-363 | MIMAT0000707 | 236 | hsa-miR-4770 | MIMAT0019924 |
| 195 | hsa-miR-365b-3p | MIMAT0022834 | 237 | hsa-miR-4771 | MIMAT0019925 |
| 196 | hsa-miR-365b-5p | MIMAT0022833 | 238 | hsa-miR-4772-3p | MIMAT0019927 |
| 197 | hsa-miR-367 | MIMAT0000719 | 239 | hsa-miR-4772-5p | MIMAT0019926 |
| 198 | hsa-miR-3676-5p | MIMAT0022734 | 240 | hsa-miR-4773 | MIMAT0019928 |
| 199 | hsa-miR-369-3p | MIMAT0000721 | 241 | hsa-miR-4774-3p | MIMAT0019930 |
| 200 | hsa-miR-369-5p | MIMAT0001621 | 242 | hsa-miR-4774-5p | MIMAT0019929 |
| 201 | hsa-miR-370 | MIMAT0000722 | 243 | hsa-miR-4775 | MIMAT0019931 |
| 202 | hsa-miR-372 | MIMAT0000724 | 244 | hsa-miR-4776-3p | MIMAT0019933 |
| 203 | hsa-miR-373 | MIMAT0000726 | 245 | hsa-miR-4776-5p | MIMAT0019932 |
| 204 | hsa-miR-374c-3p | MIMAT0022735 | 246 | hsa-miR-4777-3p | MIMAT0019935 |
| 205 | hsa-miR-375 | MIMAT0000728 | 247 | hsa-miR-4777-5p | MIMAT0019934 |
| 206 | hsa-miR-376a | MIMAT0000729 | 248 | hsa-miR-4778-3p | MIMAT0019937 |
| 207 | hsa-miR-376c | MIMAT0000720 | 249 | hsa-miR-4778-5p | MIMAT0019936 |
| 208 | hsa-miR-377 | MIMAT0000730 | 250 | hsa-miR-4779 | MIMAT0019938 |
| 209 | hsa-miR-378 | MIMAT0000732 | 251 | hsa-miR-4780 | MIMAT0019939 |
| 210 | hsa-miR-379 | MIMAT0000733 | 252 | hsa-miR-4781-3p | MIMAT0019943 |

**Supplementary Table 1** *Continued*

| # | **mature miRNA mimic** | **accession #** | **#** | **mature miRNA mimic** | **accession** |
| --- | --- | --- | --- | --- | --- |
| 253 | hsa-miR-4781-5p | MIMAT0019942 | 295 | hsa-miR-490-3p | MIMAT0002806 |
| 254 | hsa-miR-4782-3p | MIMAT0019945 | 296 | hsa-miR-490-5p | MIMAT0004764 |
| 255 | hsa-miR-4782-5p | MIMAT0019944 | 297 | hsa-miR-494 | MIMAT0002816 |
| 256 | hsa-miR-4783-3p | MIMAT0019947 | 298 | hsa-miR-496 | MIMAT0002818 |
| 257 | hsa-miR-4783-5p | MIMAT0019946 | 299 | hsa-miR-498 | MIMAT0002824 |
| 258 | hsa-miR-4784 | MIMAT0019948 | 300 | hsa-miR-4999-3p | MIMAT0021018 |
| 259 | hsa-miR-4785 | MIMAT0019949 | 301 | hsa-miR-4999-5p | MIMAT0021017 |
| 260 | hsa-miR-4786-3p | MIMAT0019955 | 302 | hsa-miR-499a-3p | MIMAT0019898 |
| 261 | hsa-miR-4786-5p | MIMAT0019954 | 303 | hsa-miR-499a-5p | MIMAT0019897 |
| 262 | hsa-miR-4787-3p | MIMAT0019957 | 304 | hsa-miR-5000-3p | MIMAT0021020 |
| 263 | hsa-miR-4787-5p | MIMAT0019956 | 305 | hsa-miR-5000-5p | MIMAT0021019 |
| 264 | hsa-miR-4788 | MIMAT0019958 | 306 | hsa-miR-5001-3p | MIMAT0021022 |
| 265 | hsa-miR-4789-3p | MIMAT0019960 | 307 | hsa-miR-5001-5p | MIMAT0021021 |
| 266 | hsa-miR-4789-5p | MIMAT0019959 | 308 | hsa-miR-5002-3p | MIMAT0021024 |
| 267 | hsa-miR-4790-3p | MIMAT0019962 | 309 | hsa-miR-5002-5p | MIMAT0021023 |
| 268 | hsa-miR-4790-5p | MIMAT0019961 | 310 | hsa-miR-5003-3p | MIMAT0021026 |
| 269 | hsa-miR-4791 | MIMAT0019963 | 311 | hsa-miR-5003-5p | MIMAT0021025 |
| 270 | hsa-miR-4792 | MIMAT0019964 | 312 | hsa-miR-5004-3p | MIMAT0021028 |
| 271 | hsa-miR-4793-3p | MIMAT0019966 | 313 | hsa-miR-5004-5p | MIMAT0021027 |
| 272 | hsa-miR-4793-5p | MIMAT0019965 | 314 | hsa-miR-5006-3p | MIMAT0021034 |
| 273 | hsa-miR-4794 | MIMAT0019967 | 315 | hsa-miR-5006-5p | MIMAT0021033 |
| 274 | hsa-miR-4795-3p | MIMAT0019969 | 316 | hsa-miR-5007-3p | MIMAT0021036 |
| 275 | hsa-miR-4795-5p | MIMAT0019968 | 317 | hsa-miR-5007-5p | MIMAT0021035 |
| 276 | hsa-miR-4796-3p | MIMAT0019971 | 318 | hsa-miR-5008-3p | MIMAT0021040 |
| 277 | hsa-miR-4796-5p | MIMAT0019970 | 319 | hsa-miR-5008-5p | MIMAT0021039 |
| 278 | hsa-miR-4797-3p | MIMAT0019973 | 320 | hsa-miR-5009-3p | MIMAT0021042 |
| 279 | hsa-miR-4797-5p | MIMAT0019972 | 321 | hsa-miR-5009-5p | MIMAT0021041 |
| 280 | hsa-miR-4798-3p | MIMAT0019975 | 322 | hsa-miR-5010-3p | MIMAT0021044 |
| 281 | hsa-miR-4798-5p | MIMAT0019974 | 323 | hsa-miR-5010-5p | MIMAT0021043 |
| 282 | hsa-miR-4799-3p | MIMAT0019977 | 324 | hsa-miR-5011-3p | MIMAT0021046 |
| 283 | hsa-miR-4799-5p | MIMAT0019976 | 325 | hsa-miR-5011-5p | MIMAT0021045 |
| 284 | hsa-miR-4800-3p | MIMAT0019979 | 326 | hsa-miR-5047 | MIMAT0020541 |
| 285 | hsa-miR-4800-5p | MIMAT0019978 | 327 | hsa-miR-506-5p | MIMAT0022701 |
| 286 | hsa-miR-4801 | MIMAT0019980 | 328 | hsa-miR-5087 | MIMAT0021079 |
| 287 | hsa-miR-4802-3p | MIMAT0019982 | 329 | hsa-miR-5088 | MIMAT0021080 |
| 288 | hsa-miR-4802-5p | MIMAT0019981 | 330 | hsa-miR-5089 | MIMAT0021081 |
| 289 | hsa-miR-4803 | MIMAT0019983 | 331 | hsa-miR-5090 | MIMAT0021082 |
| 290 | hsa-miR-4804-3p | MIMAT0019985 | 332 | hsa-miR-5091 | MIMAT0021083 |
| 291 | hsa-miR-4804-5p | MIMAT0019984 | 333 | hsa-miR-5092 | MIMAT0021084 |
| 292 | hsa-miR-483-3p | MIMAT0002173 | 334 | hsa-miR-5093 | MIMAT0021085 |
| 293 | hsa-miR-487a | MIMAT0002178 | 335 | hsa-miR-509-3p | MIMAT0002881 |
| 294 | hsa-miR-487b | MIMAT0003180 | 336 | hsa-miR-5094 | MIMAT0021086 |

**Supplementary Table 1** *Continued*

| # | **mature miRNA mimic** | **accession #** | **#** | **mature miRNA mimic** | **accession** |
| --- | --- | --- | --- | --- | --- |
| 337 | hsa-miR-5095 | MIMAT0020600 | 379 | hsa-miR-520d-5p | MIMAT0002855 |
| 338 | hsa-miR-509-5p | MIMAT0004779 | 380 | hsa-miR-520e | MIMAT0002825 |
| 339 | hsa-miR-5096 | MIMAT0020603 | 381 | hsa-miR-520f | MIMAT0002830 |
| 340 | hsa-miR-510 | MIMAT0002882 | 382 | hsa-miR-520g | MIMAT0002858 |
| 341 | hsa-miR-5100 | MIMAT0022259 | 383 | hsa-miR-521 | MIMAT0002854 |
| 342 | hsa-miR-512-5p | MIMAT0002822 | 384 | hsa-miR-522 | MIMAT0002868 |
| 343 | hsa-miR-513a-5p | MIMAT0002877 | 385 | hsa-miR-523 | MIMAT0002840 |
| 344 | hsa-miR-513c-3p | MIMAT0022728 | 386 | hsa-miR-524-3p | MIMAT0002850 |
| 345 | hsa-miR-514a-5p | MIMAT0022702 | 387 | hsa-miR-526a | MIMAT0002845 |
| 346 | hsa-miR-515-3p | MIMAT0002827 | 388 | hsa-miR-526b | MIMAT0002835 |
| 347 | hsa-miR-515-5p | MIMAT0002826 | 389 | hsa-miR-527 | MIMAT0002862 |
| 348 | hsa-miR-516a-5p | MIMAT0004770 | 390 | hsa-miR-539-3p | MIMAT0022705 |
| 349 | hsa-miR-516b | MIMAT0002859 | 391 | hsa-miR-548ab | MIMAT0018928 |
| 350 | hsa-miR-517a | MIMAT0002852 | 392 | hsa-miR-548ac | MIMAT0018938 |
| 351 | hsa-miR-517b | MIMAT0002857 | 393 | hsa-miR-548ad | MIMAT0018946 |
| 352 | hsa-miR-517c | MIMAT0002866 | 394 | hsa-miR-548ae | MIMAT0018954 |
| 353 | hsa-miR-5186 | MIMAT0021116 | 395 | hsa-miR-548ag | MIMAT0018969 |
| 354 | hsa-miR-5187-3p | MIMAT0021118 | 396 | hsa-miR-548ah | MIMAT0018972 |
| 355 | hsa-miR-5187-5p | MIMAT0021117 | 397 | hsa-miR-548ah-3p | MIMAT0020957 |
| 356 | hsa-miR-5188 | MIMAT0021119 | 398 | hsa-miR-548ai | MIMAT0018989 |
| 357 | hsa-miR-5189 | MIMAT0021120 | 399 | hsa-miR-548aj | MIMAT0018990 |
| 358 | hsa-miR-518a | MIMAT0005457 | 400 | hsa-miR-548aj-5p | MIMAT0022739 |
| 359 | hsa-miR-518b | MIMAT0002844 | 401 | hsa-miR-548ak | MIMAT0019013 |
| 360 | hsa-miR-518c | MIMAT0002848 | 402 | hsa-miR-548al | MIMAT0019024 |
| 361 | hsa-miR-518e | MIMAT0002861 | 403 | hsa-miR-548am | MIMAT0019076 |
| 362 | hsa-miR-518f | MIMAT0002842 | 404 | hsa-miR-548am-5p | MIMAT0022740 |
| 363 | hsa-miR-5190 | MIMAT0021121 | 405 | hsa-miR-548an | MIMAT0019079 |
| 364 | hsa-miR-5191 | MIMAT0021122 | 406 | hsa-miR-548ao-3p | MIMAT0021030 |
| 365 | hsa-miR-5192 | MIMAT0021123 | 407 | hsa-miR-548ao-5p | MIMAT0021029 |
| 366 | hsa-miR-5193 | MIMAT0021124 | 408 | hsa-miR-548ap-3p | MIMAT0021038 |
| 367 | hsa-miR-5194 | MIMAT0021125 | 409 | hsa-miR-548ap-5p | MIMAT0021037 |
| 368 | hsa-miR-5195-3p | MIMAT0021127 | 410 | hsa-miR-548aq-3p | MIMAT0022264 |
| 369 | hsa-miR-5195-5p | MIMAT0021126 | 411 | hsa-miR-548aq-5p | MIMAT0022263 |
| 370 | hsa-miR-5196-3p | MIMAT0021129 | 412 | hsa-miR-548ar-3p | MIMAT0022266 |
| 371 | hsa-miR-5196-5p | MIMAT0021128 | 413 | hsa-miR-548ar-5p | MIMAT0022265 |
| 372 | hsa-miR-5197-3p | MIMAT0021131 | 414 | hsa-miR-548as-3p | MIMAT0022268 |
| 373 | hsa-miR-5197-5p | MIMAT0021130 | 415 | hsa-miR-548as-5p | MIMAT0022267 |
| 374 | hsa-miR-519a | MIMAT0002869 | 416 | hsa-miR-548at-3p | MIMAT0022278 |
| 375 | hsa-miR-519d | MIMAT0002853 | 417 | hsa-miR-548at-5p | MIMAT0022277 |
| 376 | hsa-miR-519e* | MIMAT0002828 | 418 | hsa-miR-548au-3p | MIMAT0022292 |
| 377 | hsa-miR-520a-3p | MIMAT0002834 | 419 | hsa-miR-548au-5p | MIMAT0022291 |
| 378 | hsa-miR-520b | MIMAT0002843 | 420 | hsa-miR-548av-3p | MIMAT0022304 |

**Supplementary Table 1** *Continued*

| # | **mature miRNA mimic** | **accession #** | **#** | **mature miRNA mimic** | **accession** |
| --- | --- | --- | --- | --- | --- |
| 421 | hsa-miR-548av-5p | MIMAT0022303 | 463 | hsa-miR-561-5p | MIMAT0022706 |
| 422 | hsa-miR-548aw | MIMAT0022471 | 464 | hsa-miR-5680 | MIMAT0022468 |
| 423 | hsa-miR-548ax | MIMAT0022474 | 465 | hsa-miR-5681a | MIMAT0022469 |
| 424 | hsa-miR-548g-5p | MIMAT0022722 | 466 | hsa-miR-5681b | MIMAT0022480 |
| 425 | hsa-miR-548h-3p | MIMAT0022723 | 467 | hsa-miR-5682 | MIMAT0022470 |
| 426 | hsa-miR-548o-5p | MIMAT0022738 | 468 | hsa-miR-5683 | MIMAT0022472 |
| 427 | hsa-miR-548t-3p | MIMAT0022730 | 469 | hsa-miR-5684 | MIMAT0022473 |
| 428 | hsa-miR-548x-5p | MIMAT0022733 | 470 | hsa-miR-5685 | MIMAT0022475 |
| 429 | hsa-miR-549 | MIMAT0003333 | 471 | hsa-miR-5686 | MIMAT0022477 |
| 430 | hsa-miR-550a-3-5p | MIMAT0020925 | 472 | hsa-miR-5687 | MIMAT0022478 |
| 431 | hsa-miR-550b-2-5p | MIMAT0022737 | 473 | hsa-miR-5688 | MIMAT0022479 |
| 432 | hsa-miR-555 | MIMAT0003219 | 474 | hsa-miR-5689 | MIMAT0022481 |
| 433 | hsa-miR-556-5p | MIMAT0003220 | 475 | hsa-miR-5690 | MIMAT0022482 |
| 434 | hsa-miR-5571-3p | MIMAT0022258 | 476 | hsa-miR-5691 | MIMAT0022483 |
| 435 | hsa-miR-5571-5p | MIMAT0022257 | 477 | hsa-miR-5692a | MIMAT0022484 |
| 436 | hsa-miR-5572 | MIMAT0022260 | 478 | hsa-miR-5692b | MIMAT0022497 |
| 437 | hsa-miR-5579-3p | MIMAT0022270 | 479 | hsa-miR-5692c | MIMAT0022476 |
| 438 | hsa-miR-5579-5p | MIMAT0022269 | 480 | hsa-miR-5693 | MIMAT0022486 |
| 439 | hsa-miR-5580-3p | MIMAT0022274 | 481 | hsa-miR-5694 | MIMAT0022487 |
| 440 | hsa-miR-5580-5p | MIMAT0022273 | 482 | hsa-miR-5695 | MIMAT0022488 |
| 441 | hsa-miR-5581-3p | MIMAT0022276 | 483 | hsa-miR-5696 | MIMAT0022489 |
| 442 | hsa-miR-5581-5p | MIMAT0022275 | 484 | hsa-miR-5697 | MIMAT0022490 |
| 443 | hsa-miR-5582-3p | MIMAT0022280 | 485 | hsa-miR-5698 | MIMAT0022491 |
| 444 | hsa-miR-5582-5p | MIMAT0022279 | 486 | hsa-miR-5699 | MIMAT0022492 |
| 445 | hsa-miR-5583-3p | MIMAT0022282 | 487 | hsa-miR-5700 | MIMAT0022493 |
| 446 | hsa-miR-5583-5p | MIMAT0022281 | 488 | hsa-miR-5701 | MIMAT0022494 |
| 447 | hsa-miR-5584-3p | MIMAT0022284 | 489 | hsa-miR-5702 | MIMAT0022495 |
| 448 | hsa-miR-5584-5p | MIMAT0022283 | 490 | hsa-miR-5703 | MIMAT0022496 |
| 449 | hsa-miR-5585-3p | MIMAT0022286 | 491 | hsa-miR-5704 | MIMAT0022498 |
| 450 | hsa-miR-5585-5p | MIMAT0022285 | 492 | hsa-miR-5705 | MIMAT0022499 |
| 451 | hsa-miR-5586-3p | MIMAT0022288 | 493 | hsa-miR-570-5p | MIMAT0022707 |
| 452 | hsa-miR-5586-5p | MIMAT0022287 | 494 | hsa-miR-5706 | MIMAT0022500 |
| 453 | hsa-miR-5587-3p | MIMAT0022290 | 495 | hsa-miR-5707 | MIMAT0022501 |
| 454 | hsa-miR-5587-5p | MIMAT0022289 | 496 | hsa-miR-5708 | MIMAT0022502 |
| 455 | hsa-miR-5588-3p | MIMAT0022296 | 497 | hsa-miR-579 | MIMAT0003244 |
| 456 | hsa-miR-5588-5p | MIMAT0022295 | 498 | hsa-miR-584 | MIMAT0003249 |
| 457 | hsa-miR-5589-3p | MIMAT0022298 | 499 | hsa-miR-584-3p | MIMAT0022708 |
| 458 | hsa-miR-5589-5p | MIMAT0022297 | 500 | hsa-miR-621 | MIMAT0003290 |
| 459 | hsa-miR-5590-3p | MIMAT0022300 | 501 | hsa-miR-642a-3p | MIMAT0020924 |
| 460 | hsa-miR-5590-5p | MIMAT0022299 | 502 | hsa-miR-642b-5p | MIMAT0022736 |
| 461 | hsa-miR-5591-3p | MIMAT0022302 | 503 | hsa-miR-644b-3p | MIMAT0022272 |
| 462 | hsa-miR-5591-5p | MIMAT0022301 | 504 | hsa-miR-644b-5p | MIMAT0022271 |

**Supplementary Table 1** *Continued*

| # | **mature miRNA mimic** | **accession #** |
| --- | --- | --- |
| 505 | hsa-miR-652 | MIMAT0003322 |
| 506 | hsa-miR-652-5p | MIMAT0022709 |
| 507 | hsa-miR-656 | MIMAT0003332 |
| 508 | hsa-miR-659-5p | MIMAT0022710 |
| 509 | hsa-miR-660 | MIMAT0003338 |
| 510 | hsa-miR-660-3p | MIMAT0022711 |
| 511 | hsa-miR-663 | MIMAT0003326 |
| 512 | hsa-miR-668 | MIMAT0003881 |
| 513 | hsa-miR-676 | MIMAT0018204 |
| 514 | hsa-miR-720 | MIMAT0005954 |
| 515 | hsa-miR-744 | MIMAT0004945 |
| 516 | hsa-miR-760 | MIMAT0004957 |
| 517 | hsa-miR-762 | MIMAT0010313 |
| 518 | hsa-miR-766-5p | MIMAT0022714 |
| 519 | hsa-miR-873-3p | MIMAT0022717 |
| 520 | hsa-miR-888* | MIMAT0004917 |
| 521 | hsa-miR-889 | MIMAT0004921 |
| 522 | hsa-miR-9 | MIMAT0000441 |
| 523 | hsa-miR-9* | MIMAT0000442 |
| 524 | hsa-miR-92a | MIMAT0000092 |
| 525 | hsa-miR-92b | MIMAT0003218 |
| 526 | hsa-miR-93 | MIMAT0000093 |
| 527 | hsa-miR-95 | MIMAT0000094 |
| 528 | hsa-miR-96 | MIMAT0000095 |
| 529 | hsa-miR-96* | MIMAT0004510 |
| 530 | hsa-miR-98 | MIMAT0000096 |
| 531 | hsa-miR-99a | MIMAT0000097 |
| 532 | hsa-miR-99b | MIMAT0000689 |
|  |  |  |
|  |  |  |
|  |  |  |
|  |  |  |
|  |  |  |
|  |  |  |
|  |  |  |
|  |  |  |
|  |  |  |
|  |  |  |
|  |  |  |
|  |  |  |
|  |  |  |
|  |  |  |

**Supplementary table 2** Sequence of primers for DNA construction and mutagenesis

| **Primer** |  |  | **sequence (5' to 3')** |
| --- | --- | --- | --- |
| PAK2 3'UTR-1 | wt | forward | TCTAGAGAGAAGACTAATAATC |
|  |  | reverse | GAATTCACTGGTTATTATATTA |
|  | mut | forward | AGTGCCACTACCTTCTTCCGGGCTTTCCCCCTTCAATTTGG |
|  |  | reverse | CCAAATTGAAGGGGGAAAGCCCGGAAGAAGGTAGTGGCACT |
| PAK2 3'UTR-2 | wt | forward | TCTAGAAACTGGTTTATTTCTG |
|  |  | reverse | GAATTCTGTATTACTTTTAAGA |
|  | mut | forward | ATGTACTCAGAGGCACTTCCGGGCTAAGTCAAAGACCATCCTC |
|  |  | reverse | GAGGATGGTCTTTGACTTAGCCCGGAAGTGCCTCTGAGTACAT |
| CCND3 3'UTR | wt | forward | CTCGAGCCACCATGGTGAGCAA |
|  |  | reverse | AGATCTTACTTGTACAGCTCGT |
|  | mut | forward | GGGTGGGGTCATGCCGGGCTCTCCCATTGTCCC |
|  |  | reverse | GGGACAATGGGAGAGCCCGGCATGACCCCACCC |
